# Forward Genetics-Based Approaches to Understanding the Systems Biology and Molecular Mechanisms of Epilepsy

**DOI:** 10.3390/ijms24065280

**Published:** 2023-03-09

**Authors:** Anton D. Shevlyakov, Tatiana O. Kolesnikova, Murilo S. de Abreu, Elena V. Petersen, Konstantin B. Yenkoyan, Konstantin A. Demin, Allan V. Kalueff

**Affiliations:** 1 Graduate Program in Bioinformatics and Genomics, Sirius University of Science and Technology, 354340 Sochi, Russia; 2 Neuroscience Program, Sirius University of Science and Technology, 354340 Sochi, Russia; 3 Moscow Institute of Physics and Technology, 11730 Moscow, Russia; 4 Neuroscience Laboratory of COBRAIN Center for Fundamental Brain Research, and Biochemistry Department, Yerevan State Medical University named after M. Heratsi, Yerevan 0025, Armenia; 5 Institute of Translational Biomedicine, St. Petersburg State University, 199034 St. Petersburg, Russia; 6 Institute of Experimental Medicine, Almazov National Medical Research Centre, Ministry of Healthcare of Russian Federation, 194021 St. Petersburg, Russia; 7 Laboratory of Preclinical Bioscreening, Granov Russian Research Center of Radiology and Surgical Technologies, Ministry of Healthcare of Russian Federation, 197758 Pesochny, Russia; 8 Neuroscience Group, Ural Federal University, 620002 Ekaterinburg, Russia; 9 Laboratory of Biopsychiatry, Scientific Research Institute of Physiology and Basic Medicine, 630117 Novosibirsk, Russia

**Keywords:** epilepsy, genes, genetic bases, molecular network, in silico modeling

## Abstract

Epilepsy is a highly prevalent, severely debilitating neurological disorder characterized by seizures and neuronal hyperactivity due to an imbalanced neurotransmission. As genetic factors play a key role in epilepsy and its treatment, various genetic and genomic technologies continue to dissect the genetic causes of this disorder. However, the exact pathogenesis of epilepsy is not fully understood, necessitating further translational studies of this condition. Here, we applied a computational in silico approach to generate a comprehensive network of molecular pathways involved in epilepsy, based on known human candidate epilepsy genes and their established molecular interactors. Clustering the resulting network identified potential key interactors that may contribute to the development of epilepsy, and revealed functional molecular pathways associated with this disorder, including those related to neuronal hyperactivity, cytoskeletal and mitochondrial function, and metabolism. While traditional antiepileptic drugs often target single mechanisms associated with epilepsy, recent studies suggest targeting downstream pathways as an alternative efficient strategy. However, many potential downstream pathways have not yet been considered as promising targets for antiepileptic treatment. Our study calls for further research into the complexity of molecular mechanisms underlying epilepsy, aiming to develop more effective treatments targeting novel putative downstream pathways of this disorder.

## 1. Introduction

Epilepsy is a widespread, severely debilitating and complex neurological disorder characterized by central nervous system (CNS) hyperactivity, epileptic seizures and a general imbalance of excitatory and inhibitory neurotransmission. Caused by multiple external and internal factors that range from genetic mutations to infections, birth defects, stroke, and traumatic brain injuries [1], epilepsy is highly prevalent globally, with nearly 70 million people affected worldwide [2]. Furthermore, despite recent progress in antiepileptic drug development, their efficacy remains low due to various treatment-resistant types of epilepsy and multiple reported side effects (e.g., memory loss, neurotoxicity, suicides and even mortality) [3].

Genetic factors play an important role in the pathogenesis of epilepsy and modulating its sensitivity to antiepileptic therapy [1,4]. The application of genetic and genomic technologies has tremendously facilitated the discovery of genetic causes of epilepsy. For example, genome-wide association studies (GWAS) have identified multiple genes as risks of developing epilepsy, linking specific low-level somatic mutations to intractable epilepsy [5,6,7,8]. However, the exact pathogenesis of epilepsy and its true complexity remain poorly understood, necessitating further studies based on forward genetics, and more holistic, systems biology approaches in both clinical and preclinical models [9]. As such, our improved understanding of the complex molecular machinery underlying epilepsy is critical for its treatment and prevention. To better understand the complexity of molecular mechanisms involved in epilepsy, here we applied computational in silico approaches to generate a common network of molecular pathways involved in epilepsy, based on comprehensive analyses of human candidate epilepsy genes and their known molecular interactors.

## 2. Generation of a Global Molecular Network for Epilepsy

To generate a comprehensive network of molecular interactions, we analyzed multiple established human epilepsy-related genes identified previously [5] and also added new relevant information with searches performed on the Online Mendelian Inheritance in Man database (OMIM, www.omim.org/, accessed on 22 November 2022), using “epilepsy/epilepsy encephalopathy/epileptic” as search terms. We then used several publicly available genetic resources, including the Gene Cards database (www.genecards.org/, accessed on 22 November 2022) and searching publications since 1996 relevant to clinical epilepsy (search terms “epilepsy/epileptic” + gene name) in PubMed database (www.ncbi.nlm.nih.gov/pubmed/, accessed on 22 November 2022), collectively yielding a comprehensive list of 623 candidate epilepsy-linked genes (Supplementary Table S1 online) whose associated phenotypes show high variability in epilepsy and other clinical manifestations. Our analyses excluded several genes described as “functional categories of the neurodevelopment-associated epilepsy genes” [5], but not reconfirmed as associated with epilepsy in PubMed and OMIM databases. Using the generated list of putative epilepsy-associated genes, we next constructed their global molecular interaction network (based on known protein-protein interactions, PPIs) in order to identify potential key interactors that can contribute to epilepsy pathogenesis.

Finally, clustering these molecular networks based on known molecular interactions between the protein products of identified genes allowed us to identify critical molecular pathways associated with epilepsy. For this, we used the search tool for the retrieval of interacting genes/protein database (STRING version 11.5; available online: www.string-db.org, accessed on 22 November 2022) with medium confidence interval (0.40) selected for building the molecular network. To link all main clusters and identify novel potential epilepsy-associated genes, we added the number of interactors (20 interactors for the “1st shell” and 60 for the “2nd shell”) to the network settings, chosen here to ensure sufficient interconnectedness between the main clusters within the overall PPI network.

**Figure 1:**
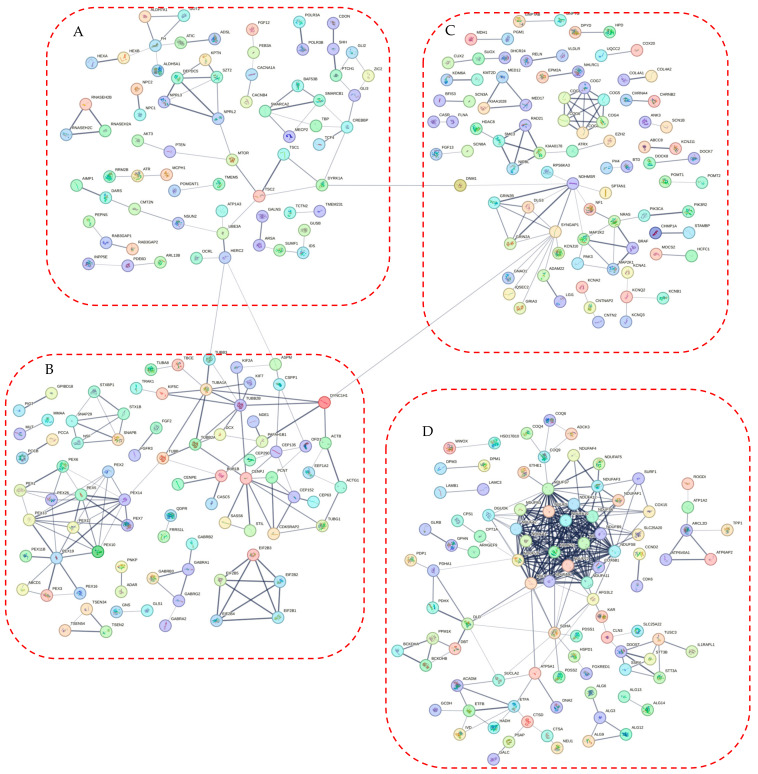
The protein-protein interaction (PPI) network obtained and visualized using the STRING database. Nodes denote individual proteins, thickness of edges represents their respective interaction scores. The network was divided into four panels (**A**–**D**); continued on the next pages, for convenience of visual presentation. Panel A mostly represents products of the mTOR system, chromatin remodeling, zinc fingers clusters and others intermediate genes. Panel B contains mainly cytoskeletal, cell division, peroxisomal, gamma-aminobutyric acid (GABA)-ergic and translation elongation initiation gene products. Panel C consists mainly of glutamate receptor- and potassium channel-related proteins. Panel D represents mitochondrial and N-oligosaccharyl transferase gene clusters.

This approach has generated a list of additional 74 putative genes likely positioned within epilepsy-associated pathways, 29 of which have been reconfirmed by published findings in PubMed, hence confirming their clinical role in epilepsy. The present study employed a conservative approach to selecting molecular interactors, using only “experimental data” as the search criterion (i.e., not assessing indirect evidence, such as text mining, co-occurrence or co-expression data) to generate a comprehensive molecular PPI network (Figure 1).

**Figure 2:**
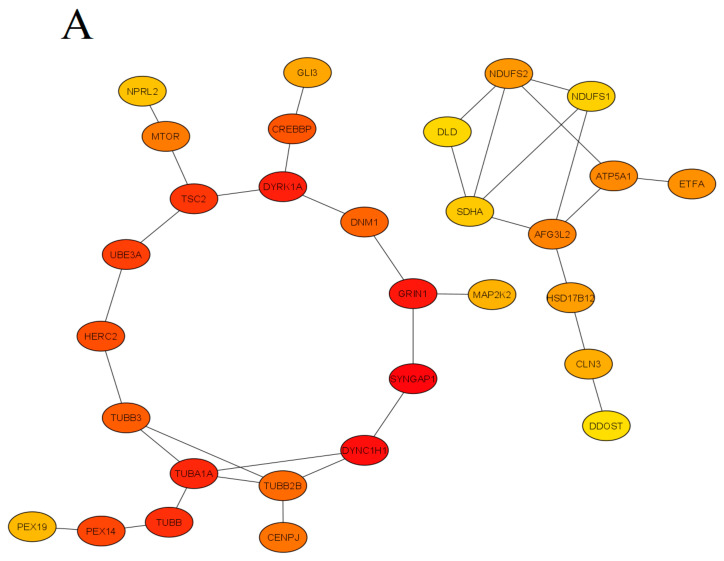

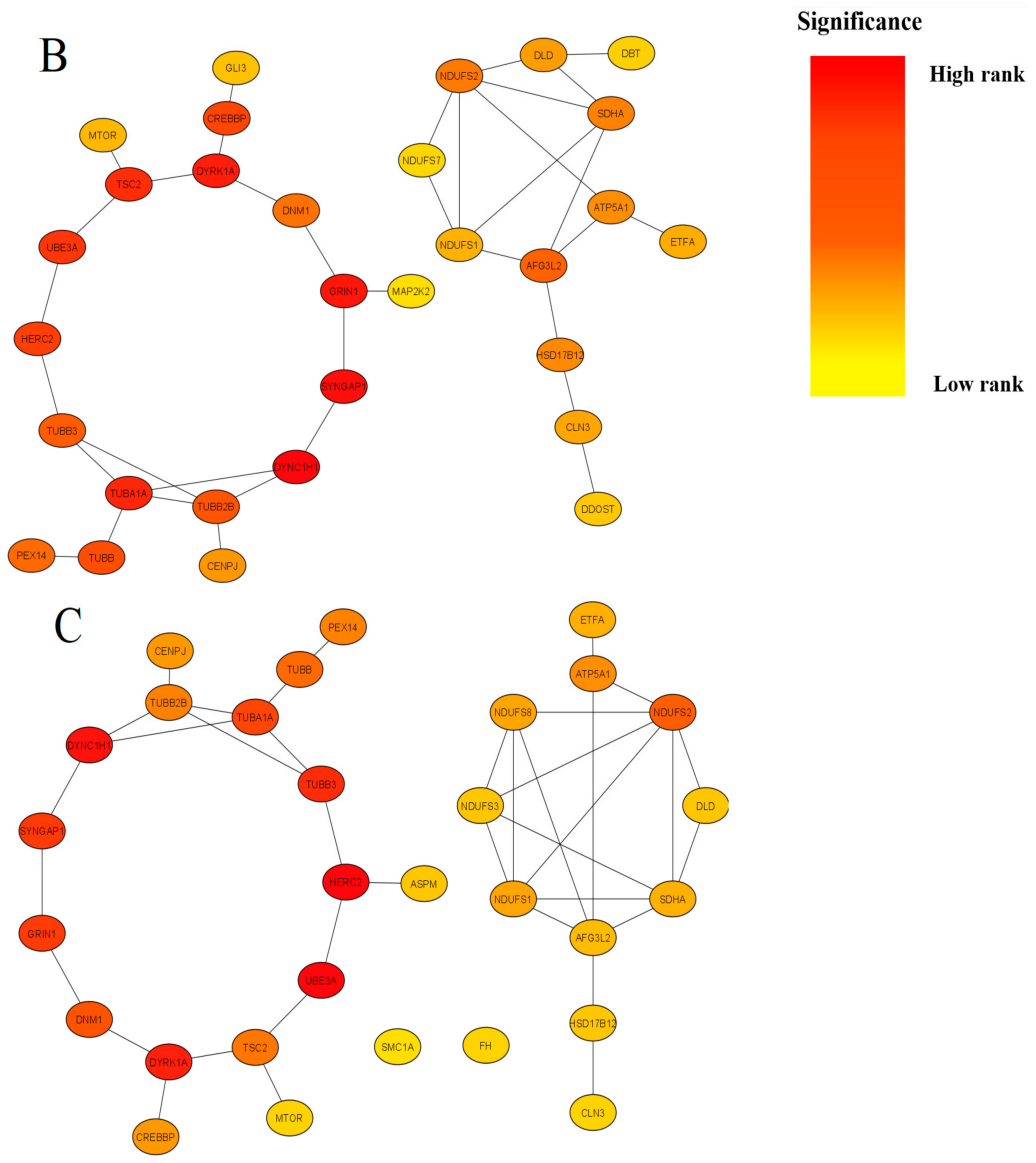
Summary of modules extracted from the global protein-protein interaction (PPI) network based on their Betweenness centrality (Panel (**A**), 30 nodes and 35 edges), Stress centrality (Panel (**B**), 30 nodes and 36 edges) and BottleNeck centrality (Panel (**C**), 30 nodes and 38 edges). Colors denote significance (high-ranked modules = red, low-ranked modules = yellow). For interpretation of the references to color in this figure legend, the reader is referred to the web version of this article.

Finally, the STRING database linked 329 of 623 into a single molecular network, leaving 240 genes that did not link to other genes and 89 genes that formed two- or three-gene clusters unlinked to the main built molecular network. The results of our analyses were next visualized using the STRING tools, as well as the CytoHubba plugin of the Cytoscape (version 3.9.1) software, searching for ‘hub’ genes from the global PPI network, as assessed by a combination of Betweenness, Stress and BottleNeck methods (Figure 2). Specifically, genes were deemed ‘hub’ if highly ranked by all three methods, which were chosen here for their known best performance in global network-based analyses from hub proteins from clusters of heterogeneous networks [10]. Betweenness centrality measures the number of times a node lies on the shortest path between other nodes. Betweenness identifies the nodes acting as “bridges” between nodes in a network, analyzing all its shortest paths and then counting how many times each node falls on one. The BottleNeck algorithm similarly searches for the shortest path between the nodes, albeit computing the minimum weight edge in the shortest path. The Stress of a node in a protein-signaling network represents the relevance of a protein as functionally capable of holding together communicating PPI nodes (its higher values reflect higher relevance of a protein for connecting regulatory molecules).

**Figure 3:**
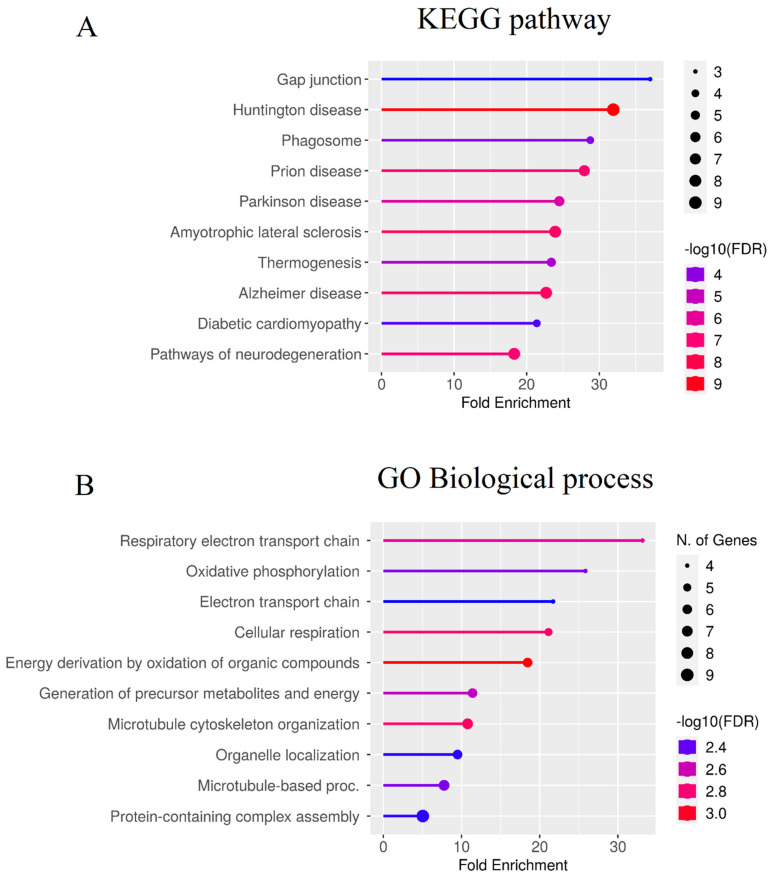
Enrichment analysis of the ‘hub’ genes identified in the present study. Color represents the -log10 (false discovery rate) of the pathway/terms, the X axis represents the enrichment, as assessed for top 10 genes by the KEGG (Kyoto Encyclopedia of Genes and Genomes) pathway (**A**) and GO (Gene Ontology) terms (**B**). For interpretation of the references to color in this figure legend, the reader is referred to the web version of this article.

To identify significantly enriched molecular pathways, we applied GO biological process and KEGG pathway enrichment analyses of hub genes (Figure 3), using the ShinyGO (www.bioinformatics.sdstate.edu/go/, accessed on 15 February 2022) version 0.77 tool with adjusted *p*-value < 0.05 considered as statistically significant.

## 3. Discussion of Identified Pathways

**Figure 4:**
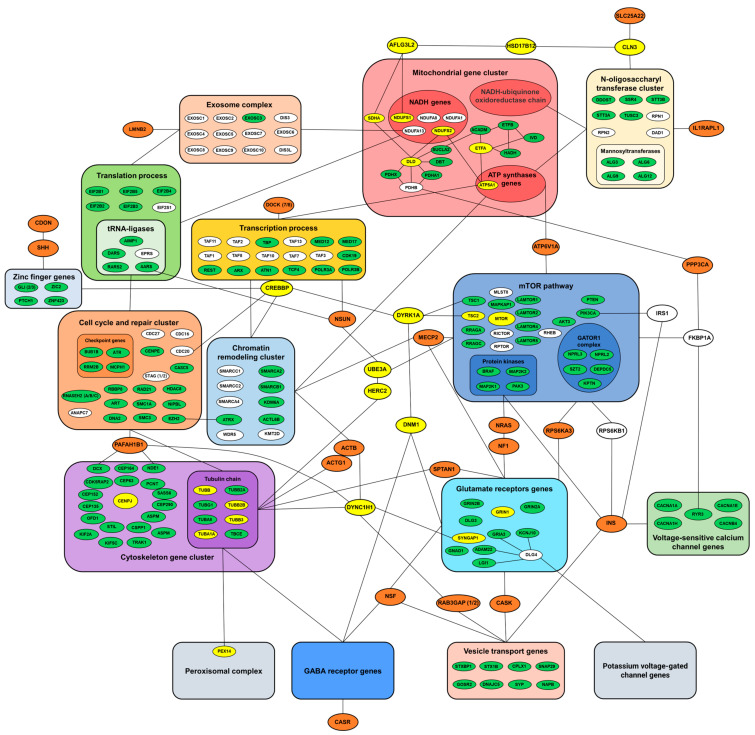
Summary diagram mapping main biomolecular pathways involved in epilepsy pathogenesis. Known biological interactions between protein products of various epilepsy-related genes are presented as bigger clusters, with selected key proteins highlighted in green, and identified central ‘hub’ genes (interconnected with most genes and clusters) in yellow. Genes originally not associated with epilepsy are shown in white, and genes not forming clusters highlighted in orange. For interpretation of the references to color in this figure legend, the reader is referred to the web version of this article.

Epilepsy pathogenesis is traditionally linked to neuronal hyperactivation that arises from aberrant ion channel (especially, Ca^++^ and K^+^) activity, imbalanced excitatory and inhibitory neurotransmission, or shifting the number of excitatory vs. inhibitory neurons [1,2,11], also see Figure 4 further.

Figure 4 shows that genes related to all these three processes have been successfully identified and mapped in the present study, hence corroborating their critical role within a complex molecular PPI network underlying epilepsy pathogenesis. However, our analyses yielded some other gene clusters beyond neural hyperactivity (Figure 4), including genes that have not been directly related to epilepsy, but are otherwise crucial for CNS functioning, hence meriting further scrutiny.

In addition, topological and functional enrichment analyses were performed using the STRING database and the ‘Network Analyzer’ function of the Cytoscape (version 3.9.1) software. For statistical analysis, we used the network without additional molecular interactors (to avoid skewing the results). Network statistics generated by the STRING database revealed a final graph containing 623 nodes and 560 edges. The average node degree and average local clustering coefficient of the network was determined to be 1.8 and 0.382, respectively (Table 1). The ‘Network Analyzer’ function estimated several other topological parameters, such as network diameter, radius, shortest path, characteristic path length and average number of neighbors (Table 1).

Utilizing the three algorithms of the cytoHubba plugin, we calculated the top 30 hub genes for each algorithm (Figure 2) and then merged their results in order to choose genes overlapping for all three methods (Table 2). The GO biological process and KEGG pathway enrichment analyses, performed by the ShinyGO (www.bioinformatics.sdstate.edu/go/, accessed on 15 February 2022, version 0.77) tool, showed that most proteins significantly enriched in established biological processes were involved in the electron transport chain, cellular respiration, cellular respiration, microtubule cytoskeleton organization and protein-containing complex assembly (Figure 3B). The KEGG enrichment analysis revealed the identified hub genes as associated with key neurodegenerative (e.g., Huntington’s, Parkinson’s and Alzheimer’s) diseases often comorbid with epilepsy [12,13]. Interestingly, significant over-enrichment was seen here for the gap junction pathway and temperature regulation (Figure 3A). The former has been consistently linked to epilepsy [14,15,16], whereas the latter can reflect increased metabolism commonly seen in epileptic phenotypes [17].

### 3.1. Mitochondrial and Metabolic Genes

Mitochondrial and metabolic genes formed one of the largest PPI clusters in the present study (Figure 4, Table 3). Although mitochondrial encephalopathies often present epileptic symptoms clinically [18], mitochondria-related epilepsy is commonly caused by mutations of mitochondrial DNA [19]. Such deficits usually affect tissues with high energy needs, including the brain, hence resulting in epilepsy when brain metabolism is disturbed. However, based on our systems biology-based analyses (Figure 4), mutations of the mitochondrial genome can impact other, higher-level systems that may also be relevant to epilepsy pathogenesis. Indeed, since many molecular processes are ATP-dependent, if a mutation occurs in a mitochondrial gene, there is a high risk of disrupting such ATP-dependent mechanisms in general. For example, as can be seen in Figure 4, the mitochondrial complex is directly related to many other cellular systems and processes, such as the exosomal complex, the mTOR signaling, the N-oligosaccharyltransferase cluster, chromatin remodeling, as well as transcription and translation factors, whose functional activity depends on normally functioning mitochondria, and may therefore be disrupted by mutations in mitochondrial genes.

Directly linked to them, the exosomal complex genes are also involved in the maturation and degradation of various types of RNA, and thereby can play an important role in epigenetic regulation. The exosomal complex modulates the activity of mitochondrial genes, regulating their expression using microRNAs [20]. In addition, the disruption of exosomal activity is itself a powerful trigger for epilepsy, negatively affecting many systems, such as the mTOR system and the translation machinery [21,22]. Separately from this complex, the *LMNB2* gene mutations are often accompanied by epilepsy [23], and this gene is also involved in epigenetic regulation, directly affecting chromatin and the structure of the nucleus [24].

Genes of the N-oligosaccharyltransferase complex are crucial for cell development and survival. Congenital glycosylation disorders (CDG) are a heterogeneous group of congenital metabolic diseases with multisystem clinical lesions [25,26,27,28] due to mutations in N-linked glycosylation genes, that may also affect CNS and, thus, contribute to epilepsy [29]. Multiple mannosyltransferase genes are also located in this gene cluster (Figure 4), and their aberrant activity is associated with a very rare subtype of CDG, accompanied by several forms of early-onset epileptic encephalopathies [30,31]. Finally, together with the mitochondrial compartment, the N-oligosaccharyltransferase genes are associated with such important genes as *CLN3* and *SLC25A22*, responsible for the formation and transport of endosomes and glutamate, a major excitatory neurotransmitter [32,33,34,35] directly involved in epilepsy pathogenesis.

### 3.2. The mTOR Signaling Pathway

The mammalian target of rapamycin (mTOR) pathway is a key signaling system regulating cell growth, development, proliferation and motility. Like mitochondrial genes, mutations within the mTOR pathway genes are the commonest cause of epilepsy, often accompanying focal cortical dysplasia (PCD) and other cortical malformations [8,11,36]. MTOR functions as a serine/threonine protein kinase forming two main complexes, mTORC1 and mTORC2. MTOR acts as a protein tyrosine kinase that promotes the activation of insulin receptors and insulin-like growth factor receptors [37]. Since mTORC2 is also involved in the control and maintenance of cytoskeleton [38], this system is key for neuroplasticity and, accordingly, the distribution of inhibitory and excitatory neurons that, as already mentioned, are directly related to epilepsy pathogenesis. Mutations in the mTOR-inhibiting (e.g., tuberous sclerosis TSC1, TSC2 and GATOR1 complex) genes are particularly strongly linked to epilepsy. For example, hyperactivation of the mTORC1 complex and the rise of S6 and S6K phosphorylation [11,39,40,41] produce enlarged neurons, which, in turn, lead to neurotransmitter imbalance and focal seizures.

Notably, the mTOR system is associated with glutamate signaling, Ca^++^ genes and the mitochondrial compartment (Figure 2). Furthermore, the mTOR pathway is controlled by multiple other mechanisms, including the methyl CpG-binding protein 2 gene (*MECP2*), an epigenetic regulator with several important functions in the brain [42]. De novo mutations of X-linked *MECP2* are the main cause of Rett syndrome often involving epileptic symptoms [43]. *MECP2* mutations in humans with Rett syndrome are associated with impaired regulation of nucleolin, rRNA transcripts, and mTOR signaling through participation in post-transcriptional processing of certain microRNAs [44,45].

Another important mTOR regulator involved in epilepsy is dual specificity tyrosine-phosphorylation-regulated kinase 1A (DYRK1A), an inhibitor of mTORC1. In contrast, its overexpression increases phosphorylation and activity of both TSC1 and TSC2, whereas increased phosphorylation of S6K1 and 4E-BP1 is observed in *DYRK1A* knockdown cancer cells—the effect inhibited by the mTOR-inhibiting drug rapamycin [46,47]. A deficiency in ubiquitin protein ligase E3A (UBE3A) also modulates the mTOR system activity, elevating levels of TSC2 responsible for inhibiting mTOR, hence hyperactivating the mTORC1-S6K1 pathway [48]. Its link to Ca^++^ channels is also relevant here, since Ca^++^ channelopathies themselves often cause epilepsy, and mutations in such channel genes also impact the mTOR system. For example, mutations in *FKBP1A* are associated with RYR3 dysregulation [49], whereas mutations of *CACNA1A* impair mTOR signaling [50].

### 3.3. Transcription Factors and Chromatin Remodeling Genes

As shown in Figure 4, genes of the mTOR pathway, such as *MECP2* and *DYRK1A*, also interact with other genes, including the gene of the CREB binding protein (CREBBP), a critical cellular epigenetic regulator and a common transcription factor that specifically binds to DNA upstream of the 5′ ends of genes to initiate the landing of RNA polymerase, thereby exerting its regulatory effects. Although some tumor-related transcription factors can participate in the pathogenesis of neurological diseases, the transcription factor genes have not been viewed as classical epilepsy-associated genes, and their putative role in epilepsy merits further scrutiny [51]. In the present study, *CREBBP* has emerged as one of the central hub genes of the generated epilepsy PPI network (Figure 1). Not surprisingly, mounting evidence implicates CREBBP in multiple physiological processes, such as cell cycle regulation, neuroplasticity, learning, memory [52,53] and, more recently, epilepsy [54,55]. CREBBP is also an important regulator of the brain-derived neurotrophic factor (BDNF), indirectly affecting the mTOR pathway [56] and, hence, epileptogenesis. 

REST (RE1 silencing transcription factor) is an important transcriptional repressor that silences target genes through epigenetic remodeling, thereby regulating neurogenesis, differentiation and the expression of specific genes controlling brain development. REST, like CREBBP, regulates numerous target genes that encode neuronal receptors, ion channels, neuropeptides and synaptic proteins, key for synaptic plasticity and vesicular transport [52,54,57]. Not surprisingly, REST and CREBBP are both prominently present in the epilepsy PPI network generated here (Figure 1). In addition to transcription factors, this network also contains zinc finger and chromatin remodeling factors (CRFs). Although zinc finger genes have not been recognized as directly linked to epilepsy, they are important modulators of the transcription process and are involved in the sonic hedgehog signaling pathway that is directly associated with epilepsy [58,59,60,61]. As such, our analyses suggest zinc finger genes as novel potential candidate epilepsy genes. 

CRFs play a crucial role in epigenetic regulation, determining the activity of transcription factors by forming open sections of DNA for their landing. Chromatin remodeling is an ATP- and actin-dependent process, and may therefore be directly linked to the mitochondrial and cytoskeleton gene clusters [62] implicated in epilepsy by our analyses (Figure 1). Interestingly, among multiple CRF genes, only *SMARCA2*, *SMARCB1*, *ACTL6B* and *KDM5C* have been previously associated with epilepsy, and some other members of this cluster (e.g., *SMARCC1*, *SMARCC2*, *SMARCA4* and *WRD5*) are only cursorily mentioned among epilepsy candidate genes [62,63,64,65]. As such, our analyses suggest that CRFs may represent a more important group of putative epileptic genes than previously recognized, thereby calling for further probing of the role of these genes in epilepsy in both clinical and preclinical models.

### 3.4. Cytoskeleton and Cell Division

In epileptic brain, cytoskeletal disruption is often viewed as being secondary to aberrant neuronal activity. However, mounting data indicate that cytoskeletal and cell division genes are critical factors in the pathogenesis of epilepsy, as well as neuronal migration disorders and channelopathies [66,67]. The cell cycle genes are also involved in neuronal migration and proliferation, and are closely related to cytoskeletal function as well. For instance, commonly causing epilepsy, mutations in tubulin coding genes [68,69] are responsible for a wide range of brain malformations secondary to abnormal neuronal migration, manifesting as motor disorders, mental retardation and epilepsy [70]. Moreover, tubulin is an important protein for the transport of the gamma aminobutyric acid A (GABA-A) receptors and for formation of peroxisomes [69,71,72]. Collectively, this suggests that aberrant cytoskeletal functions may cause epilepsy indirectly, impacting major CNS transport systems, including the formation of both key membrane receptors and cell growth and mobility.

### 3.5. Some Other Potential Novel Epilepsy-Associated Genes 

Our in silico analyses have identified 74 additional genes that are actively involved either in processes within the same cluster, or interact between different clusters (Table 3). Although these genes all represent important components of the clusters they form, there is either no confirmation of their direct involvement in epilepsy, or they remain unstudied in this regard. By identifying these genes as core elements of epilepsy-related clusters that form a meaningful molecular network (Figure 4), the present study calls for further in-depth analyses of such novel potential candidate genes and their putative predicted role in epilepsy.

## 4. Concluding Remarks

An important aspect of the present in silico study is its focus on epilepsy-associated proteins using unbiased bioinformatics-based analyses of known molecular interactions. Overall, this supports the involvement of cytoskeletal, mitochondrial and metabolic pathways in epilepsy, which until recently have been considered secondary to the core of its pathogenesis. Although mutations in transcription factors-, zinc finger-, or chromatin remodeling-related genes may not directly cause neuronal hyperactivity and epilepsy, they may still disrupt cellular processes that could trigger a wide range of consequences, indirectly evoking epileptic symptoms.

We also recognize the fact that most modern antiepileptic therapies demonstrate low effectiveness, as they usually tend to target a single ‘terminal’ key mechanisms of epilepsy. For example, common antiepileptic drugs target GABA-A receptors (e.g., benzodiazepines, vigabatrin and phenobarbital) and Ca^++^ channels (ethosuximide), without affecting downstream cellular processes. However, as recent studies show, the true root cause of a disorder often lays within the common downstream pathways responsible for the operation of the entire system as a whole. 

Although novel medications have already been proposed for some of them (e.g., antiepileptic activity of rapamycin that acts by suppressing the mTOR signaling system), the majority of other potential downstream pathways are not yet considered as feasible targets. In turn, this may also impede adequate diagnostics and treatment (e.g., in mitochondrial encephalopathy, as with ordinary epilepsy, symptomatic seizures are observed, but some classical antiepileptic drugs, such as valproate, would typically only worsen the situation) [73,74]. Thus, increasingly deeper understanding of genetic causes underlying both common and rare forms of epilepsy, involving a wider spectrum of molecular events and clusters (Figure 4), as well as their interplay, and deeper downstream common signaling processes, are urgently needed for tackling epilepsy and identifying novel targets and drugs for its treatment.

## Figures and Tables

**Table 1:** Topological parameters of the protein-protein interaction (PPI) network generated in the present study.

Source	Network Statistics	Values
STRING	Number of nodes	623
(Including single nodes)	Number of edges	560
Network Analyzer	Average node degree	1.8
(Not including single nodes)	Avg. local clustering coefficient	0.382
	Expected number of edges	176
	PPI enrichment *p*-value	<1 × 10^−16^
	Number of nodes	329
	Number of edges	560
	Avg. number of neighbors	3.404
	Network diameter	6
	Network radius	1
	Characteristic path lengths	1.936
	Clustering coefficient	0.158
	Network density	0.005

**Table 2:** Functional analyses of the identified ‘hub’ genes.

№	Genes	Details
1	*MTOR*	Serine/threonine-protein kinase mTOR
2	*PEX14*	Peroxisomal membrane protein PEX14
3	*NDUFS2*	NADH dehydrogenase [ubiquinone] iron-sulfur protein 2
4	*SDHA*	
5	*TUBB*	Succinate dehydrogenase [ubiquinone] flavoprotein subunit, mitochondrial
6	*DLD*	Tubulin beta chain
7	*DNM1*	Dihydrolipoyl dehydrogenase, mitochondrial
8	*GRIN1*	Dynamin-1
9	*HSD17B12*	Glutamate receptor ionotropic, NMDA 1
10	*TUBA1A*	Very-long-chain 3-oxoacyl-CoA reductase
11	*CENPJ*	Tubulin alpha-1A chain
12	*DYNC1H1*	Centromere protein J
13	*UBE3A*	Cytoplasmic dynein 1 heavy chain 1
14	*ETFA*	Ubiquitin-protein ligase E3A
15	*TSC2*	Electron transfer flavoprotein subunit alpha, mitochondrial
16	*CREBBP*	CREB-binding protein
17	*CLN3*	CLN3 lysosomal/endosomal transmembrane protein
18	*TUBB3*	Tubulin beta-3 chain
19	*AFG3L2*	AFG3-like protein 2
20	*ATP5A1*	ATP synthase complex subunit B1, mitochondrial
21	*DYRK1A*	Dual specificity tyrosine-phosphorylation-regulated kinase 1A
22	*HERC2*	E3 ubiquitin-protein ligase HERC2
23	*NDUFS1*	NADH-ubiquinone oxidoreductase 75 kDa subunit, mitochondrial
24	*SYNGAP1*	Ras/Rap GTPase-activating protein SynGAP
25	*TUBB2B*	Tubulin beta-2B chain

**Table 3:** Epilepsy-related genes analyzed in the present study and their clustering within a complex molecular network. Bolded genes listed here were absent in the original list of epileptic genes selected for our analyses here (see above), but have been identified by the STRING database, hence representing potential novel molecular targets.

Molecular Function/Cluster	Genes
Mitochondrial genes	*GCDH*, *COQ4*, *COQ9*, *COQ6*, *PQBP1*, *ATP5A1*, ***NDUFA6***, *NDUFA2*, ***NDUFB6***, *SURF1*, *NDUFV1*, ***NDUFA5***, ***NDUFS5***, *COX10*, *ADCK3*, *NDUFAF5*, ***NDUFB8***, *PDSS1*, *AFG3L2*, *SDHAF1*, *BCKDHB*, *COX6B1*, *NDUFS2*, *NDUFB9*, *NDUFA11*, ***NDUFB10***, *DBT*, ***NDUFB1***, *NDUFAF1*, *NDUFAF3*, *CPS1*, ***NDUFB4***, ***NDUFA13***, ***SUCLG1***, *NDUFS1*, *NDUFA9*, *DLD*, *NDUFS7*, *COX15*, *NDUFS6*, *PDSS2*, ***PDHB***, *DGUOK*, ***NDUFAB1***, *HSPD1*, ***MT-CO2***, *NDUFA12*, *NDUFB3*, *PPM1K*, *NDUFS3*, *PDHA1*, *NDUFB5*, ***SDHB***, *HADH*, *CPT1A*, ***NDUFV3***, *SUCLA2*, *NDUFAF4*, *NDUFA7*, ***MT-ND6***, ***MT-ND5***, ***MT-ND4***, ***UQCRFS1***, *PDP1*, ***NDUFB7***, *COX20*, *ETHE1*, *IVD*, *BCKDHA*, *NDUFA10*, *PDHX*, *FOXRED1*, ***NDUFA8***, ***MT-ND1***, *ATP6V0A1*, *AΤΡ6AΡ2*, *AΤΡ1A2*, ***ATP6V1D***, *ATP6V1A*
Exosomal genes	***EXOSC6***, ***EXOSC4***, ***DIS3L***, ***EXOSC2***, ***DIS3***, ***EXOSC1***, ***EXOSC10***, ***EXOSC5***, ***EXOSC9***, ***EXOSC8***, *EXOSC3*, ***EXOSC7***, ***SKIV2L2***
N-oligosaccharyl transferase-related genes	*DDOST*, *STT3A*, *ALG12*, ***RPN2***, *PC*, *ALG9*, *STT3B*, *SSR4*, ***DAD1***, *TUSC3*, *ALG6*, ***RPN1***, *ALG3*
The mTOR signaling pathway	*MAP2K1*, *RRAGC*, *LAMTOR5*, *MAP2K2*, *MECP2*, *NPRL2*, *DEPDC5*, *TSC1*, ***RHEB***, *LAMTOR1*, *MTOR*, *SZT2*, *PAK3*, *BRAF*, *MAPKAP1*, ***RICTOR***, ***MLST8***, *PTEN*, ***RPTOR***, *PIK3CA*, *TSC2*, *KPTN*, *LAMTOR2*, *RRAGA*, *LAMTOR4*, *NPRL3*, *AKT3*
Transcription process	*CDK19*, *TCF4*, *TAF13*, *CREBBP*, *MED12*, *ARS*, ***TAF10***, *POLR3B*, *TBP*, ***TAF7***, *ATN1*, *REST*, ***TAF1***, ***TAF2***, ***TAF3***, ***TAF4***, ***TAF4B***, ***TAF5***, ***TAF6***, ***TAF9B***, ***TAF11***, ***TAF12***, ***TAF8***, *MED17*, *POLR3A*, ***GTF2A1***, ***GTF2A2***, ***AK6***
Chromatin remodeling	***SMARCC1***,***SMARCC2***, ***SMARCC2***, *SMARCB1*, ***SMARCA4***, *ACTL6B*, ***WDR5***, *KDM6A*, *KMT2D*, *ATRX*
Translation process	*EIF2B1*, *EIF2B5*, *EIF2B4*, *EIF2B2*, *EIF2B3*, ***EIF2S1***, *AIMP1*, *DARS*, ***EPRS***, *RARS2*, *AARS*, *EEF1A2*
Glutamate neurotransmission	*GRIN1*, *GRIN2A*, *SYNGAP1*, *DLG3*, *GRIN2B*, *KCNJ10*, *GNAO1*, *GRIA3*, *ADAM22*, ***DLG4***, *LGI1*, *GABBR2*
Cytoskeleton and cell cycle	*TUBA1A*, *RNASEH2B*, *STILL*, *ACTB*, *SMC1A*, *RNASEH2A*, *TUBB2B*, *KIF7*, *RNASEH2C*, *ATR*, *NIPBL*, *TRACK1*, *TUBB2A*, *FGF13*, *CEP135*, *CEP152*, *BUB1B*, *PAFAH1B1*, *SASS6*, *DNA2*, *CSPP1*, *CASC5*, *SMC3*, *TUBA8*, *ASPM*, *CEP290*, *EZH2*, *CEP63*, *TUBB*, *KIF2A*, *ACTG1*, *TUBG1*, *DCX*, *CENPE*, *MCPH1*, *OFD1*, *TUBB3*, *CDK5RAP2*, *ANAPC7*, *HDAC8*, *PCNT*, *CENPJ*, *MAD2L1*, *KIF5C*, *NDE1*, *RAD21*, ***CDC20***, *TBCE*, *RRM2B*, *DYNC1H1*, ***STAG1***, ***STAG2***, ***CDC27***, ***CDC16***
Peroxisomal complex	*PEX5*, *PEX2*, *PEX7*, *PEX6*, *PEX3*, *PEX12*, *ABCD1*, *PEY*, *PEX16*, *PEX14*, *PEX26*, *PEX19*, *PEX11B*, *PEX13*, *PEX1*
Gamma aminobutyric acid (GABA) neurotransmission	*GABRA1*, *GABRA2*, *GABRG2*, *GABRA5*, *GABRB3*, *GABRA5*, *GABRB2*, *GABRD*
Vesicular transport	*STXBP1*, *STX1B*, *CPLX1*, *SNAP29*, *GOSR2*, *DNAJC5*, *SYP*, *NAPB*
Potassium voltage-gated channel genes	*KCNA2*, *KCNA1*, *CNTNAP2*, *CNTN2*, *KCNQ2*, *KCNB1*, *KCNQ3*
Voltage-sensitive Ca^++^ channel function	*CACNA1A*, *CACNA1H*, *CACNA1E*, *CACNB4*, *RYR3*
Golgi complex	*COG6*, *COG7*, *COG1*, *COG5*, *COG8*, *COG4*
Genes not included in specific molecular clusters	*SLC25A22*, *LMNB2*, *DOCK7 DOCK8*, *NSUN*, *PAFAH1B1*, *DYRK1A*, *UBE3A*, *HERC2*, *DNM1*, *RPS6KA3*, *RAB3GAP1*, *RAB3GAP2*, *CASK*, *CASR*, *INS*, *PPP3CA*, *ATP6*, *IL1RAPL1*, *HSD17B12*, *CLN3*, *ALG3L2*, *GLI2*, *GLI3*, *PTCH1*, *ZIC2*, *SHH*, *CDON*, ***FKBP1A***, ***IRS1***, *RPS6KA3*, ***RPS6KB1***

## Data Availability

Not applicable.

## Supplementary Materials

The following supporting information can be downloaded at: https://www.mdpi.com/article/10.3390/ijms24065280/s1.
