# Supplementary material for: Forward Genetics-Based Approaches to Understanding the Systems Biology and Molecular Mechanisms of Epilepsy
Source: Int J Mol Sci. 2023 Mar 9;24(6):5280. doi: 10.3390/ijms24065280 (PMC10049737; doi:10.3390/ijms24065280)
Supplement: Supplementary file 1 [file ijms-24-05280-s001.zip › ijms-2167964-supplementary.pdf]

Supplementary Table S1 online. Selected genes forming a common molecular pathway of epilepsy (Figure 1) generated in the present study

Genes	Annotations based on the STRING database
GCDH	Glutaryl-CoA dehydrogenase, mitochondrial; Catalyzes the oxidative decarboxylation of glutaryl-CoA to crotonyl-CoA and CO(2) in the degradative pathway of L-lysine, L-hydroxylysine, and L-tryptophan metabolism.
COQ4	Ubiquinone biosynthesis protein COQ4 homolog, mitochondrial; Component of the coenzyme Q biosynthetic pathway. May play a role in organizing a multi-subunit COQ enzyme complex required for coenzyme Q biosynthesis.
COQ9	Ubiquinone biosynthesis protein COQ9, mitochondrial; Lipid-binding protein involved in the biosynthesis of coenzyme Q, also named ubiquinone, an essential lipid-soluble electron transporter for aerobic cellular respiration. Binds a phospholipid of at least 10 carbons in each acyl group.
COQ6	Ubiquinone biosynthesis monooxygenase COQ6, mitochondrial; FAD-dependent monooxygenase required for the C5-ring hydroxylation during ubiquinone biosynthesis. Catalyzes the hydroxylation of 3-polyprenyl-4-hydroxybenzoic acid to 3- polyprenyl-4,5-dihydroxybenzoic acid.
PQBP1	Polyglutamine-binding protein 1; Intrinsically disordered protein that acts as a scaffold, and which is involved in pre-mRNA splicing, transcription regulation, innate immunity and neuron development. Interacts with splicing-related factors via the intrinsically disordered region and regulates alternative splicing of target pre-mRNA species. May suppress the ability of POU3F2 to transactivate the DRD1 gene in a POU3F2 dependent manner. Can activate transcription directly or via association with the transcription machinery
UQCRC2	Cytochrome b-c1 complex subunit 2, mitochondrial; This is a component of the ubiquinol-cytochrome C reductase complex, part of the mitochondrial respiratory chain. The core protein 2 is required for the assembly of the complex; M16 metallopeptidases
ATP5A1	ATP synthase subunit alpha, mitochondrial; Mitochondrial membrane ATP synthase (F(1)F(0) ATP synthase or Complex V) produces ATP from ADP in the presence of a proton gradient across the membrane which is generated by electron transport complexes of the respiratory chain. F-type ATPases consist of two structural domains, F(1) - containing the extramembraneous catalytic core, and F(0) - containing the membrane proton channel, linked together
NDUFA6	NADH dehydrogenase [ubiquinone] 1 alpha subcomplex subunit 6; Accessory subunit of the mitochondrial membrane respiratory chain NADH dehydrogenase (Complex I), that is believed to be not involved in catalysis. Complex I functions in the transfer of electrons from NADH to the respiratory chain.
NDUFA2	NADH dehydrogenase [ubiquinone] 1 alpha subcomplex subunit 2; Accessory subunit of the mitochondrial membrane respiratory chain NADH dehydrogenase (Complex I), that is believed not to be involved in catalysis. Complex I functions in the transfer of electrons from NADH to the respiratory chain. The immediate electron acceptor for the enzyme is believed to be ubiquinone
NDUFB6	NADH dehydrogenase [ubiquinone] 1 beta subcomplex subunit 6; Accessory subunit of the mitochondrial membrane respiratory chain NADH dehydrogenase (Complex I), that is believed not to be involved in catalysis. Complex I functions in the transfer of electrons from NADH to the respiratory chain. The immediate electron acceptor for the enzyme is believed to be ubiquinone
SURF1	SURF1, cytochrome c oxidase assembly factor; Surfeit locus protein 1; Component of the MITRAC (mitochondrial translation regulation assembly intermediate of cytochrome c oxidase complex) complex, that regulates cytochrome c oxidase assembly
CYC1	Cytochrome c1, heme protein, mitochondrial; This is the heme-containing component of the cytochrome b-c1 complex, which accepts electrons from Rieske protein and transfers electrons to cytochrome c in the mitochondrial respiratory chain; Apoptosome
NDUFV1	NADH dehydrogenase [ubiquinone] flavoprotein 1, mitochondrial; Core subunit of the mitochondrial membrane respiratory chain NADH dehydrogenase (Complex I) that is believed to belong to the minimal assembly required for catalysis. Complex I functions in the transfer of electrons from NADH to the respiratory chain.
NDUFA5	NADH dehydrogenase [ubiquinone] 1 alpha subcomplex subunit 5; Accessory subunit of the mitochondrial membrane respiratory chain NADH dehydrogenase (Complex I), that is believed not to be involved in catalysis. Complex I functions in the transfer of electrons from NADH to

the respiratory chain. The immediate electron acceptor for the enzyme is believed to be ubiquinone

NDUFS5 NADH dehydrogenase [ubiquinone] iron-sulfur protein 5; Accessory subunit of the mitochondrial membrane respiratory chain NADH dehydrogenase (Complex I), that is believed not to be involved in catalysis. Complex I functions in the transfer of electrons from NADH to the respiratory chain. The immediate electron acceptor for the enzyme is believed to be ubiquinone

COX10 Protoheme IX farnesyltransferase, mitochondrial; Converts protoheme IX and farnesyl diphosphate to heme O; Mitochondrial respiratory chain complex assembly factors

ADCK3 Atypical kinase COQ8A, mitochondrial; Atypical kinase involved in the biosynthesis of coenzyme Q, also named ubiquinone, an essential lipid-soluble electron transporter for aerobic cellular respiration. Its substrate specificity is unclear: does not show any protein kinase activity. Probably acts as a small molecule kinase, possibly a lipid kinase that phosphorylates a prenyl lipid in the ubiquinone biosynthesis pathway, as suggested by its ability to bind coenzyme Q lipid intermediates. Shows an unusual selectivity for binding ADP over ATP

NDUFAF5 Nadh dehydrogenase [ubiquinone] 1 alpha subcomplex assembly factor 5; Arginine-hydroxylase NDUFAF5, mitochondrial; Arginine hydroxylase involved in the assembly of mitochondrial NADH:ubiquinone oxidoreductase complex (complex I, MT-ND1) at early stages. Acts by mediating hydroxylation of 'Arg-111' of NDUFS7. May also have methyltransferase activity (Probable)

NDUFB8 NADH dehydrogenase [ubiquinone] 1 beta subcomplex subunit 8, mitochondrial; Accessory subunit of the mitochondrial membrane respiratory chain NADH dehydrogenase (Complex I), that is believed not to be involved in catalysis. Complex I functions in the transfer of electrons from NADH to the respiratory chain. The immediate electron acceptor for the enzyme is believed to be ubiquinone

PDSS1 Decaprenyl-diphosphate synthase subunit 1; Supplies decaprenyl diphosphate, the precursor for the side chain of the isoprenoid quinones ubiquinone-10; Belongs to the FPP/GGPP synthase family

AFG3L2 AFG3-like protein 2; ATP-dependent protease which is essential for axonal and neuron development. In neurons, mediates degradation of SMDT1/EMRE before its assembly with the uniporter complex, limiting the availability of SMDT1/EMRE for MCU assembly and promoting efficient assembly of gatekeeper subunits with MCU. Required for the maturation of paraplegin (SPG7) after its cleavage by mitochondrial-processing peptidase (MPP), converting it into a proteolytically active mature form (By similarity); In the N-terminal section; belongs to the AAA ATPase family

SDHAF1 Succinate dehydrogenase assembly factor 1, mitochondrial; Plays an essential role in the assembly of succinate dehydrogenase (SDH), an enzyme complex (also referred to as respiratory complex II) that is a component of both the tricarboxylic acid (TCA) cycle and the mitochondrial electron transport chain, and which couples the oxidation of succinate to fumarate with the reduction of ubiquinone (coenzyme Q) to ubiquinol. Promotes maturation of the iron-sulfur protein subunit SDHB of the SDH catalytic dimer, protecting it from the deleterious effects of oxidants

BCKDHB 2-oxoisovalerate dehydrogenase subunit beta, mitochondrial; The branched-chain alpha-keto dehydrogenase complex catalyzes the overall conversion of alpha-keto acids to acyl-CoA and CO(2). It contains multiple copies of three enzymatic components: branched-chain alpha-keto acid decarboxylase (E1), lipoamide acyltransferase (E2) and lipoamide dehydrogenase (E3)

COX6B1 Cytochrome c oxidase subunit 6B1; Connects the two COX monomers into the physiological dimeric form; Mitochondrial complex IV: cytochrome c oxidase subunits

NDUFS2 NADH dehydrogenase [ubiquinone] iron-sulfur protein 2, mitochondrial; Core subunit of the mitochondrial membrane respiratory chain NADH dehydrogenase (Complex I) that is believed to belong to the minimal assembly required for catalysis. Complex I functions in the transfer of electrons from NADH to the respiratory chain. The immediate electron acceptor for the enzyme is believed to be ubiquinone

NDUFB9 NADH dehydrogenase [ubiquinone] 1 beta subcomplex subunit 9; Accessory subunit of the mitochondrial membrane respiratory chain NADH dehydrogenase (Complex I), that is believed to be not involved in catalysis. Complex I functions in the transfer of electrons from NADH to the respiratory chain. The immediate electron acceptor for the enzyme is believed to be ubiquinone

NDUFA11 NADH dehydrogenase [ubiquinone] 1 alpha subcomplex subunit 11; Accessory subunit of the mitochondrial membrane respiratory chain NADH dehydrogenase (Complex I), that is believed not to be involved in catalysis. Complex I functions in the transfer of electrons from NADH to the respiratory chain. The immediate electron acceptor for the enzyme is believed to be ubiquinone; NADH:ubiquinone oxidoreductase supernumerary subunits

NDUFB10	NADH dehydrogenase [ubiquinone] 1 beta subcomplex subunit 10; Accessory subunit of the mitochondrial membrane respiratory chain NADH dehydrogenase (Complex I), that is believed not to be involved in catalysis. Complex I functions in the transfer of electrons from NADH to the respiratory chain. The immediate electron acceptor for the enzyme is believed to be ubiquinone; NADH:ubiquinone oxidoreductase supernumerary subunits
DBT	2-oxoisovalerate dehydrogenase E2 component (dihydrolipoyl transacylase); Dihydrolipoamide branched chain transacylase E2; The branched-chain alpha-keto dehydrogenase complex catalyzes the overall conversion of alpha-keto acids to acyl-CoA and CO(2). It contains multiple copies of three enzymatic components: branched-chain alpha-keto acid decarboxylase (E1), lipoamide acyltransferase (E2) and lipoamide dehydrogenase (E3)
NDUFB1	NADH dehydrogenase [ubiquinone] 1 beta subcomplex subunit 1; Accessory subunit of the mitochondrial membrane respiratory chain NADH dehydrogenase (Complex I) that is believed not to be involved in catalysis. Complex I functions in the transfer of electrons from NADH to the respiratory chain. The immediate electron acceptor for the enzyme is believed to be ubiquinone
NDUFAF1	Nadh dehydrogenase [ubiquinone] 1 alpha subcomplex assembly factor 1; Complex I intermediate-associated protein 30, mitochondrial; Chaperone protein involved in the assembly of the mitochondrial NADH:ubiquinone oxidoreductase complex (complex I); Belongs to the CIA30 family
NDUFAF3	NADH dehydrogenase [ubiquinone] 1 alpha subcomplex assembly factor 3; Essential factor for the assembly of mitochondrial NADH:ubiquinone oxidoreductase complex (complex I)
CPS1	Carbamoyl-phosphate synthase [ammonia], mitochondrial; Involved in the urea cycle of ureotelic animals where the enzyme plays an important role in removing excess ammonia from the cell
NDUFB4	NADH dehydrogenase [ubiquinone] 1 beta subcomplex subunit 4; Accessory subunit of the mitochondrial membrane respiratory chain NADH dehydrogenase (Complex I), that is believed not to be involved in catalysis. Complex I functions in the transfer of electrons from NADH to the respiratory chain. The immediate electron acceptor for the enzyme is believed to be ubiquinone
NDUFA13	NADH dehydrogenase [ubiquinone] 1 alpha subcomplex subunit 13; Accessory subunit of the mitochondrial membrane respiratory chain NADH dehydrogenase (Complex I), that is believed not to be involved in catalysis. Complex I functions in the transfer of electrons from NADH to the respiratory chain. The immediate electron acceptor for the enzyme is believed to be ubiquinone. Involved in the interferon/all-trans-retinoic acid (IFN/RA) induced cell death. This apoptotic activity is inhibited by interaction with viral IRF1. Prevents the transactivation of STAT3 target genes
SUCLG1	Succinate--CoA ligase [ADP/GDP-forming] subunit alpha, mitochondrial; Succinyl-CoA synthetase functions in the citric acid cycle (TCA), coupling the hydrolysis of succinyl-CoA to the synthesis of either ATP or GTP and thus represents the only step of substrate-level phosphorylation in the TCA. The alpha subunit of the enzyme binds the substrates coenzyme A and phosphate, while succinate binding and specificity for either ATP or GTP is provided by different beta subunits
NDUFS1	NADH-ubiquinone oxidoreductase 75 kDa subunit, mitochondrial; Core subunit of the mitochondrial membrane respiratory chain NADH dehydrogenase (Complex I) that is believed to belong to the minimal assembly required for catalysis. Complex I functions in the transfer of electrons from NADH to the respiratory chain. The immediate electron acceptor for the enzyme is believed to be ubiquinone (By similarity). This is the largest subunit of complex I and it is a component of the iron-sulfur (IP) fragment of the enzyme. It may form part of the active site crevice where NADH is oxidized
NDUFA9	NADH dehydrogenase [ubiquinone] 1 alpha subcomplex subunit 9, mitochondrial; Accessory subunit of the mitochondrial membrane respiratory chain NADH dehydrogenase (Complex I), that is believed not to be involved in catalysis. Complex I functions in the transfer of electrons from NADH to the respiratory chain. The immediate electron acceptor for the enzyme is believed to be ubiquinone; NADH:ubiquinone oxidoreductase supernumerary subunits
DLD	Dihydrolipoyl dehydrogenase, mitochondrial; Lipoamide dehydrogenase is a component of the glycine cleavage system as well as an E3 component of three alpha-ketoacid dehydrogenase complexes (pyruvate-, alpha-ketoglutarate-, and branched-chain amino acid-dehydrogenase complex). In monomeric form has additional moonlighting function as serine protease. Involved in the hyperactivation of spermatazoa during capacitation and in the spermatazoal acrosome reaction
NDUFS7	NADH dehydrogenase [ubiquinone] iron-sulfur protein 7, mitochondrial; Core subunit of the mitochondrial membrane respiratory chain NADH dehydrogenase (Complex I) that is believed

	to belong to the minimal assembly required for catalysis. Complex I functions in the transfer of electrons from NADH to the respiratory chain.
COX15	Cytochrome c oxidase assembly protein COX15 homolog; May be involved in the biosynthesis of heme A; Mitochondrial respiratory chain complex assembly factors
PDSS2	Decaprenyl-diphosphate synthase subunit 2; Supplies decaprenyl diphosphate, the precursor for the side chain of the isoprenoid quinones ubiquinone-10
SLC6A5	Solute carrier family 6 (neurotransmitter transporter, glycine) member 5/9; Sodium- and chloride-dependent glycine transporter 2; Terminates the action of glycine by its high affinity sodium-dependent reuptake into presynaptic terminals. May be responsible for the termination of neurotransmission at strychnine-sensitive glycinergic synapses; Belongs to the sodium:neurotransmitter symporter (SNF) (TC 2.A.22) family. SLC6A5 subfamily
TMEM70	Transmembrane protein 70, mitochondrial; Involved in biogenesis of mitochondrial ATP synthase; Belongs to the TMEM70 family
SDHA	Succinate dehydrogenase [ubiquinone] flavoprotein subunit, mitochondrial; Flavoprotein (FP) subunit of succinate dehydrogenase (SDH) that is involved in complex II of the mitochondrial electron transport chain and is responsible for transferring electrons from succinate to ubiquinone (coenzyme Q). Can act as a tumor suppressor; Belongs to the FAD-dependent oxidoreductase 2 family. FRD/SDH subfamily
PDHB	Pyruvate dehydrogenase E1 component subunit beta, mitochondrial; The pyruvate dehydrogenase complex catalyzes the overall conversion of pyruvate to acetyl-CoA and CO(2), and thereby links the glycolytic pathway to the tricarboxylic cycle
DGUOK	Deoxyguanosine kinase, mitochondrial; Phosphorylates deoxyguanosine and deoxyadenosine in the mitochondrial matrix, with the highest efficiency for deoxyguanosine. In non-replicating cells, where cytosolic dNTP synthesis is down-regulated, mtDNA synthesis depends solely on DGUOK and TK2. Phosphorylates certain nucleoside analogs. Widely used as target of antiviral and chemotherapeutic agents
NDUFAB1	Acyl carrier protein, mitochondrial; Carrier of the growing fatty acid chain in fatty acid biosynthesis (By similarity). Accessory and non-catalytic subunit of the mitochondrial membrane respiratory chain NADH dehydrogenase (Complex I), which functions in the transfer of electrons from NADH to the respiratory chain; Belongs to the acyl carrier protein (ACP) family
HSPD1	Heat shock protein family d (hsp60) member 1; 60 kDa heat shock protein, mitochondrial; Chaperonin implicated in mitochondrial protein import and macromolecular assembly. Together with Hsp10, facilitates the correct folding of imported proteins. May also prevent misfolding and promote the refolding and proper assembly of unfolded polypeptides generated under stress conditions in the mitochondrial matrix. The functional units of these chaperonins consist of heptameric rings of the large subunit Hsp60, which function as a back-to-back double ring
MT-CO2	Mitochondrially encoded cytochrome C oxidase subunit 2; Cytochrome c oxidase is the component of the respiratory chain that catalyzes the reduction of oxygen to water. Subunits 1-3 form the functional core of the enzyme complex. Subunit 2 transfers the electrons from cytochrome C via its binuclear copper A center to the bimetallic center of the catalytic subunit 1
NDUFA12	NADH dehydrogenase [ubiquinone] 1 alpha subcomplex subunit 12; Accessory subunit of the mitochondrial membrane respiratory chain NADH dehydrogenase (Complex I), that is believed not to be involved in catalysis. Complex I functions in the transfer of electrons from NADH to the respiratory chain. The immediate electron acceptor for the enzyme is believed to be ubiquinone
NDUFB3	NADH dehydrogenase [ubiquinone] 1 beta subcomplex subunit 3; Accessory subunit of the mitochondrial membrane respiratory chain NADH dehydrogenase (Complex I), that is believed not to be involved in catalysis. Complex I functions in the transfer of electrons from NADH to the respiratory chain. The immediate electron acceptor for the enzyme is believed to be ubiquinone
PPM1K	Protein phosphatase 1K, mitochondrial; Regulates the mitochondrial permeability transition pore and is essential for cellular survival and development; Belongs to the PP2C family
NDUFS3	NADH dehydrogenase [ubiquinone] iron-sulfur protein 3, mitochondrial; Core subunit of the mitochondrial membrane respiratory chain NADH dehydrogenase (Complex I) that is believed to belong to the minimal assembly required for catalysis. Complex I functions in the transfer of electrons from NADH to the respiratory chain. The immediate electron acceptor for the enzyme is believed to be ubiquinone (By similarity); NADH:ubiquinone oxidoreductase core subunits
PDHA1	Pyruvate dehydrogenase E1 component subunit alpha, somatic form, mitochondrial; The pyruvate dehydrogenase complex catalyzes the overall conversion of pyruvate to acetyl-CoA and CO(2), and thereby links the glycolytic pathway to the tricarboxylic cycle
NDUFB5	NADH dehydrogenase [ubiquinone] 1 beta subcomplex subunit 5, mitochondrial; Accessory subunit of the mitochondrial membrane respiratory chain NADH dehydrogenase (Complex I),

	that is believed not to be involved in catalysis. Complex I functions in the transfer of electrons from NADH to the respiratory chain. The immediate electron acceptor for the enzyme is believed to be ubiquinone
SDHB	Succinate dehydrogenase [ubiquinone] iron-sulfur subunit, mitochondrial; Iron-sulfur protein (IP) subunit of succinate dehydrogenase (SDH) that is involved in complex II of the mitochondrial electron transport chain and is responsible for transferring electrons from succinate to ubiquinone (coenzyme Q)
HADH	Hydroxyacyl-coenzyme A dehydrogenase, mitochondrial; Plays an essential role in the mitochondrial beta- oxidation of short chain fatty acids. Exerts it highest activity toward 3-hydroxybutyryl-CoA; Belongs to the 3-hydroxyacyl-CoA dehydrogenase family
CPT1A	Carnitine O-palmitoyltransferase 1, liver isoform; Catalyzes the transfer of the acyl group of long-chain fatty acid-CoA conjugates onto carnitine, an essential step for the mitochondrial uptake of long-chain fatty acids and their subsequent beta-oxidation in the mitochondrion. Plays an important role in triglyceride metabolism
NDUFV3	NADH dehydrogenase [ubiquinone] flavoprotein 3, mitochondrial; Accessory subunit of the mitochondrial membrane respiratory chain NADH dehydrogenase (Complex I), that is believed not to be involved in catalysis. Complex I functions in the transfer of electrons from NADH to the respiratory chain. The immediate electron acceptor for the enzyme is believed to be ubiquinone. May be the terminally assembled subunit of Complex I
SUCLA2	Succinate--CoA ligase [ADP-forming] subunit beta, mitochondrial; ATP-specific succinyl-CoA synthetase functions in the citric acid cycle (TCA), coupling the hydrolysis of succinyl-CoA to the synthesis of ATP and thus represents the only step of substrate-level phosphorylation in the TCA. The beta subunit provides nucleotide specificity of the enzyme and binds the substrate succinate, while the binding sites for coenzyme A and phosphate are found in the alpha subunit (By similarity)
NDUFAF4	NADH dehydrogenase [ubiquinone] 1 alpha subcomplex assembly factor 4; Involved in the assembly of mitochondrial NADH:ubiquinone oxidoreductase complex (complex I). May be involved in cell proliferation and survival of hormone-dependent tumor cells. May be a regulator of breast tumor cell invasion
NDUFA7	NADH dehydrogenase [ubiquinone] 1 alpha subcomplex subunit 7; Accessory subunit of the mitochondrial membrane respiratory chain NADH dehydrogenase (Complex I), that is believed not to be involved in catalysis. Complex I functions in the transfer of electrons from NADH to the respiratory chain. The immediate electron acceptor for the enzyme is believed to be ubiquinone; NADH:ubiquinone oxidoreductase supernumerary subunits
MT-ND6	NADH-ubiquinone oxidoreductase chain 6; Core subunit of the mitochondrial membrane respiratory chain NADH dehydrogenase (Complex I) that is believed to belong to the minimal assembly required for catalysis. Complex I functions in the transfer of electrons from NADH to the respiratory chain. The immediate electron acceptor for the enzyme is believed to be ubiquinone (By similarity); NADH:ubiquinone oxidoreductase core subunits
MT-ND5	NADH-ubiquinone oxidoreductase chain 5; Core subunit of the mitochondrial membrane respiratory chain NADH dehydrogenase (Complex I) that is believed to belong to the minimal assembly required for catalysis. Complex I functions in the transfer of electrons from NADH to the respiratory chain. The immediate electron acceptor for the enzyme is believed to be ubiquinone (By similarity); NADH:ubiquinone oxidoreductase core subunits
MT-ND4	Mitochondrially encoded nadh:ubiquinone oxidoreductase core subunit 4; NADH-ubiquinone oxidoreductase chain 4; Core subunit of the mitochondrial membrane respiratory chain NADH dehydrogenase (Complex I) that is believed to belong to the minimal assembly required for catalysis. Complex I functions in the transfer of electrons from NADH to the respiratory chain. The immediate electron acceptor for the enzyme is believed to be ubiquinone (By similarity)
UQCRFS1	Ubiquinol-cytochrome c reductase, rieske iron-sulfur polypeptide 1; Cytochrome b-c1 complex subunit Rieske, mitochondrial; Cytochrome b-c1 complex subunit Rieske, mitochondrial: Component of the mitochondrial ubiquinol-cytochrome c reductase complex dimer (complex III dimer), which is a respiratory chain that generates an electrochemical potential coupled to ATP synthesis. Incorporation of UQCRFS1 is the penultimate step in complex III assembly (By similarity)
PDP1	[Pyruvate dehydrogenase [acetyl-transferring]]-phosphatase 1, mitochondrial; Catalyzes the dephosphorylation and concomitant reactivation of the alpha subunit of the E1 component of the pyruvate dehydrogenase complex; Belongs to the PP2C family
NDUFB7	NADH dehydrogenase [ubiquinone] 1 beta subcomplex subunit 7; Accessory subunit of the mitochondrial membrane respiratory chain NADH dehydrogenase (Complex I), that is believed not to be involved in catalysis. Complex I functions in the transfer of electrons from NADH to the respiratory chain. The immediate electron acceptor for the enzyme is believed to be ubiquinone

COX20	Cytochrome c oxidase protein 20 homolog; Mitochondrial respiratory chain complex assembly factors; Belongs to the COX20 family
ETHE1	Persulfide dioxygenase ETHE1, mitochondrial; Sulfur dioxygenase that plays an essential role in hydrogen sulfide catabolism in the mitochondrial matrix. Hydrogen sulfide (H(2)S) is first oxidized by SQRDL, giving rise to cysteine persulfide residues. ETHE1 consumes molecular oxygen to catalyze the oxidation of the persulfide, once it has been transferred to a thiophilic acceptor, such as glutathione (R-SSH)
IVD	Isovaleryl-CoA dehydrogenase, mitochondrial; Acyl-CoA dehydrogenase family
BCKDHA	Branched chain keto acid dehydrogenase e1, alpha polypeptide; 2-oxoisovalerate dehydrogenase subunit alpha, mitochondrial; The branched-chain alpha-keto dehydrogenase complex catalyzes the overall conversion of alpha-keto acids to acyl-CoA and CO(2). It contains multiple copies of three enzymatic components: branched-chain alpha-keto acid decarboxylase (E1), lipoamide acyltransferase (E2) and lipoamide dehydrogenase (E3)
NDUFA10	NADH dehydrogenase [ubiquinone] 1 alpha subcomplex subunit 10, mitochondrial; Accessory subunit of the mitochondrial membrane respiratory chain NADH dehydrogenase (Complex I), that is believed not to be involved in catalysis. Complex I functions in the transfer of electrons from NADH to the respiratory chain. The immediate electron acceptor for the enzyme is believed to be ubiquinone; NADH:ubiquinone oxidoreductase supernumerary subunits
PDHX	Pyruvate dehydrogenase protein X component, mitochondrial; Required for anchoring dihydrolipoamide dehydrogenase (E3) to the dihydrolipoamide transacetylase (E2) core of the pyruvate dehydrogenase complexes of eukaryotes. This specific binding is essential for a functional PDH complex
FOXRED1	FAD-dependent oxidoreductase domain-containing protein 1; Required for the assembly of the mitochondrial membrane respiratory chain NADH dehydrogenase (Complex I). Involved in mid-late stages of complex I assembly
NDUFA8	NADH dehydrogenase [ubiquinone] 1 alpha subcomplex subunit 8; Accessory subunit of the mitochondrial membrane respiratory chain NADH dehydrogenase (Complex I), that is believed not to be involved in catalysis. Complex I functions in the transfer of electrons from NADH to the respiratory chain. The immediate electron acceptor for the enzyme is believed to be ubiquinone; NADH:ubiquinone oxidoreductase supernumerary subunits
MT-ND1	NADH-ubiquinone oxidoreductase chain 1; Core subunit of the mitochondrial membrane respiratory chain NADH dehydrogenase (Complex I) that is believed to belong to the minimal assembly required for catalysis. Complex I functions in the transfer of electrons from NADH to the respiratory chain. The immediate electron acceptor for the enzyme is believed to be ubiquinone (By similarity); NADH:ubiquinone oxidoreductase core subunits
ATP6V0A1	V-type proton ATPase 116 kDa subunit a isoform 1; Required for assembly and activity of the vacuolar ATPase. Potential role in differential targeting and regulation of the enzyme for a specific organelle (By similarity); V-type ATPases
ATP6V1E1	V-type proton ATPase subunit E 1; Subunit of the peripheral V1 complex of vacuolar ATPase essential for assembly or catalytic function. V-ATPase is responsible for acidifying a variety of intracellular compartments in eukaryotic cells; V-type ATPases
ATP6V1D	V-type proton ATPase subunit D; Subunit of the peripheral V1 complex of vacuolar ATPase. Vacuolar ATPase is responsible for acidifying a variety of intracellular compartments in eukaryotic cells, thus providing most of the energy required for transport processes in the vacuolar system (By similarity). May play a role in cilium biogenesis through regulation of the transport and the localization of proteins to the cilium
ATP6V1A	V-type proton ATPase catalytic subunit A; Catalytic subunit of the peripheral V1 complex of vacuolar ATPase. V-ATPase vacuolar ATPase is responsible for acidifying a variety of intracellular compartments in eukaryotic cells. In aerobic conditions, involved in intracellular iron homeostasis, thus triggering the activity of Fe(2+) prolyl hydroxylase (PHD) enzymes, and leading to HIF1A hydroxylation and subsequent proteasomal degradation; Belongs to the ATPase alpha/beta chains family
EXOSC6	Exosome complex component MTR3; Non-catalytic component of the RNA exosome complex which has 3'->5' exoribonuclease activity and participates in a multitude of cellular RNA processing and degradation events. In the nucleus, the RNA exosome complex is involved in proper maturation of stable RNA species such as rRNA, snRNA and snoRNA, in the elimination of RNA processing by-products and non-coding 'pervasive' transcripts, such as antisense RNA species and promoter-upstream transcripts (PROMPTs), and of mRNAs with processing defects, thereby limiting or excluding their export to the cytoplasm
EXOSC4	Exosome complex component RRP41; Non-catalytic component of the RNA exosome complex which has 3'->5' exoribonuclease activity and participates in a multitude of cellular RNA processing and degradation events. In the nucleus, the RNA exosome complex is involved in proper maturation of stable RNA species such as rRNA, snRNA and snoRNA, in the

	elimination of RNA processing by-products and non-coding 'pervasive' transcripts, such as antisense RNA species and promoter-upstream transcripts (PROMPTs), and of mRNAs with processing defects, thereby limiting or excluding their export to the cytoplasm
DIS3L	DIS3-like exonuclease 1; Putative cytoplasm-specific catalytic component of the RNA exosome complex which has 3'->5' exoribonuclease activity and participates in a multitude of cellular RNA processing and degradation events. In the cytoplasm, the RNA exosome complex is involved in general mRNA turnover and specifically degrades inherently unstable mRNAs containing AU-rich elements (AREs) within their 3' untranslated regions, and in RNA surveillance pathways, preventing translation of aberrant mRNAs. It seems to be involved in degradation of histone mRNA
EXOSC2	Exosome complex component RRP4; Non-catalytic component of the RNA exosome complex which has 3'->5' exoribonuclease activity and participates in a multitude of cellular RNA processing and degradation events. In the nucleus, the RNA exosome complex is involved in proper maturation of stable RNA species such as rRNA, snRNA and snoRNA, in the elimination of RNA processing by-products and non-coding 'pervasive' transcripts, such as antisense RNA species and promoter-upstream transcripts (PROMPTs), and of mRNAs with processing defects, thereby limiting or excluding their export to the cytoplasm
DIS3	DIS3 homolog, exosome endoribonuclease and 3'-5' exoribonuclease; Exosome complex exonuclease RRP44; Putative catalytic component of the RNA exosome complex which has 3'->5' exoribonuclease activity and participates in a multitude of cellular RNA processing and degradation events. In the nucleus, the RNA exosome complex is involved in proper maturation of stable RNA species such as rRNA, snRNA and snoRNA, in the elimination of RNA processing by-products and non-coding 'pervasive' transcripts, such as antisense RNA species and promoter-upstream transcripts (PROMPTs), and of mRNAs with processing defects, thereby limiting or excluding their export to the cytoplasm
EXOSC1	Exosome complex component CSL4; Non-catalytic component of the RNA exosome complex which has 3'->5' exoribonuclease activity and participates in a multitude of cellular RNA processing and degradation events. In the nucleus, the RNA exosome complex is involved in proper maturation of stable RNA species such as rRNA, snRNA and snoRNA, in the elimination of RNA processing by-products and non-coding 'pervasive' transcripts, such as antisense RNA species and promoter-upstream transcripts (PROMPTs), and of mRNAs with processing defects, thereby limiting or excluding their export to the cytoplasm
EXOSC10	Exosome complex exonuclease rrp6; Exosome component 10; Putative catalytic component of the RNA exosome complex which has 3'->5' exoribonuclease activity and participates in a multitude of cellular RNA processing and degradation events. In the nucleus, the RNA exosome complex is involved in proper maturation of stable RNA species such as rRNA, snRNA and snoRNA, in the elimination of RNA processing by-products and non-coding 'pervasive' transcripts, such as antisense RNA species and promoter-upstream transcripts (PROMPTs), and of mRNAs with processing defects, thereby limiting or excluding their export to the cytoplasm
EXOSC5	Exosome complex component RRP46; Non-catalytic component of the RNA exosome complex which has 3'->5' exoribonuclease activity and participates in a multitude of cellular RNA processing and degradation events. In the nucleus, the RNA exosome complex is involved in proper maturation of stable RNA species such as rRNA, snRNA and snoRNA, in the elimination of RNA processing by-products and non-coding 'pervasive' transcripts, such as antisense RNA species and promoter-upstream transcripts (PROMPTs), and of mRNAs with processing defects, thereby limiting or excluding their export to the cytoplasm
EXOSC9	Exosome complex component RRP45; Non-catalytic component of the RNA exosome complex which has 3'->5' exoribonuclease activity and participates in a multitude of cellular RNA processing and degradation events. In the nucleus, the RNA exosome complex is involved in proper maturation of stable RNA species such as rRNA, snRNA and snoRNA, in the elimination of RNA processing by-products and non-coding 'pervasive' transcripts, such as antisense RNA species and promoter-upstream transcripts (PROMPTs), and of mRNAs with processing defects, thereby limiting or excluding their export to the cytoplasm
EXOSC8	Exosome complex component RRP43; Non-catalytic component of the RNA exosome complex which has 3'->5' exoribonuclease activity and participates in a multitude of cellular RNA processing and degradation events. In the nucleus, the RNA exosome complex is involved in proper maturation of stable RNA species such as rRNA, snRNA and snoRNA, in the elimination of RNA processing by-products and non-coding 'pervasive' transcripts, such as antisense RNA species and promoter-upstream transcripts (PROMPTs), and of mRNAs with processing defects, thereby limiting or excluding their export to the cytoplasm
EXOSC3	Exosome complex component RRP40; Non-catalytic component of the RNA exosome complex which has 3'->5' exoribonuclease activity and participates in a multitude of cellular RNA processing and degradation events. In the nucleus, the RNA exosome complex is involved in

	proper maturation of stable RNA species such as rRNA, snRNA and snoRNA, in the elimination of RNA processing by-products and non-coding 'pervasive' transcripts, such as antisense RNA species and promoter-upstream transcripts (PROMPTs), and of mRNAs with processing defects, thereby limiting or excluding their export to the cytoplasm
EXOSC7	Exosome complex component RRP42; Non-catalytic component of the RNA exosome complex which has 3'->5' exoribonuclease activity and participates in a multitude of cellular RNA processing and degradation events. In the nucleus, the RNA exosome complex is involved in proper maturation of stable RNA species such as rRNA, snRNA and snoRNA, in the elimination of RNA processing by-products and non-coding 'pervasive' transcripts, such as antisense RNA species and promoter-upstream transcripts (PROMPTs), and of mRNAs with processing defects, thereby limiting or excluding their export to the cytoplasm
DDOST	Dolichyl-diphosphooligosaccharide--protein glycosyltransferase 48 kDa subunit; Essential subunit of the N-oligosaccharyl transferase (OST) complex which catalyzes the transfer of a high mannose oligosaccharide from a lipid-linked oligosaccharide donor to an asparagine residue within an Asn-X-Ser/Thr consensus motif in nascent polypeptide chains. Required for the assembly of both SST3A- and SS3B-containing OST complexes. Required for efficient N-glycosylation; Glutamine amidotransferase like class 1 domain containing
STT3A	Dolichyl-diphosphooligosaccharide--protein glycosyltransferase subunit STT3A; Catalytic subunit of the N-oligosaccharyl transferase (OST) complex which catalyzes the transfer of a high mannose oligosaccharide from a lipid-linked oligosaccharide donor to an asparagine residue within an Asn-X-Ser/Thr consensus motif in nascent polypeptide chains. N-glycosylation occurs cotranslationally and the complex associates with the Sec61 complex at the channel-forming translocon complex that mediates protein translocation across the endoplasmic reticulum (ER)
MAGT1	Magnesium transporter protein 1; Acts as accessory component of the N-oligosaccharyl transferase (OST) complex which catalyzes the transfer of a high mannose oligosaccharide from a lipid-linked oligosaccharide donor to an asparagine residue within an Asn-X-Ser/Thr consensus motif in nascent polypeptide chains. Involved in N-glycosylation of STT3B-dependent substrates. Specifically required for the glycosylation of a subset of acceptor sites that are near cysteine residues; in this function seems to act redundantly with TUSC3
ALG12	Dol-P-Man:Man(7)GlcNAc(2)-PP-Dol alpha-1,6-mannosyltransferase; Adds the eighth mannose residue in an alpha-1,6 linkage onto the dolichol-PP-oligosaccharide precursor (dolichol-PP- Man(7)GlcNAc(2)) required for protein glycosylation; Belongs to the glycosyltransferase 22 family
RPN2	Dolichyl-diphosphooligosaccharide--protein glycosyltransferase subunit 2; Essential subunit of the N-oligosaccharyl transferase (OST) complex which catalyzes the transfer of a high mannose oligosaccharide from a lipid-linked oligosaccharide donor to an asparagine residue within an Asn-X-Ser/Thr consensus motif in nascent polypeptide chains; Belongs to the SWP1 family
PC	Pyruvate carboxylase, mitochondrial; Pyruvate carboxylase catalyzes a 2-step reaction, involving the ATP-dependent carboxylation of the covalently attached biotin in the first step and the transfer of the carboxyl group to pyruvate in the second. Catalyzes in a tissue specific manner, the initial reactions of glucose (liver, kidney) and lipid (adipose tissue, liver, brain) synthesis from pyruvate
ALG9	Alpha-1,2-mannosyltransferase ALG9; Catalyzes the transfer of mannose from Dol-P-Man to lipid-linked oligosaccharides; Belongs to the glycosyltransferase 22 family
STT3B	Dolichyl-diphosphooligosaccharide--protein glycosyltransferase subunit STT3B; Catalytic subunit of the N-oligosaccharyl transferase (OST) complex which catalyzes the transfer of a high mannose oligosaccharide from a lipid-linked oligosaccharide donor to an asparagine residue within an Asn-X-Ser/Thr consensus motif in nascent polypeptide chains. N-glycosylation occurs cotranslationally and the complex associates with the Sec61 complex at the channel-forming translocon complex that mediates protein translocation across the endoplasmic reticulum (ER)
SSR4	Translocon-associated protein subunit delta; TRAP proteins are part of a complex whose function is to bind calcium to the ER membrane and thereby regulate the retention of ER resident proteins; Belongs to the TRAP-delta family
DAD1	Dolichyl-diphosphooligosaccharide--protein glycosyltransferase subunit DAD1; Essential subunit of the N-oligosaccharyl transferase (OST) complex which catalyzes the transfer of a high mannose oligosaccharide from a lipid-linked oligosaccharide donor to an asparagine residue within an Asn-X-Ser/Thr consensus motif in nascent polypeptide chains. Required for the assembly of both SST3A- and SS3B-containing OST complexes. Required for efficient N-glycosylation. Loss of the DAD1 protein triggers apoptosis; Belongs to the DAD/OST2 family
TUSC3	Oligosaccharyltransferase complex subunit gamma; Tumor suppressor candidate 3; Acts as accessory component of the N-oligosaccharyl transferase (OST) complex which catalyzes the

	transfer of a high mannose oligosaccharide from a lipid-linked oligosaccharide donor to an asparagine residue within an Asn-X-Ser/Thr consensus motif in nascent polypeptide chains. Involved in N-glycosylation of STT3B-dependent substrates. Specifically required for the glycosylation of a subset of acceptor sites that are near cysteine residues; in this function seems to act redundantly with MAGT1
ALG6	Dolichyl pyrophosphate Man9GlcNAc2 alpha-1,3-glucosyltransferase; Adds the first glucose residue to the lipid-linked oligosaccharide precursor for N-linked glycosylation. Transfers glucose from dolichyl phosphate glucose (Dol-P-Glc) onto the lipid-linked oligosaccharide Man(9)GlcNAc(2)-PP-Dol; Alpha-1,3-glucosyltransferases
RPN1	Dolichyl-diphosphooligosaccharide--protein glycosyltransferase subunit 1; Essential subunit of the N-oligosaccharyl transferase (OST) complex which catalyzes the transfer of a high mannose oligosaccharide from a lipid-linked oligosaccharide donor to an asparagine residue within an Asn-X-Ser/Thr consensus motif in nascent polypeptide chains; Belongs to the OST1 family
ALG3	Dol-P-Man:Man(5)GlcNAc(2)-PP-Dol alpha-1,3-mannosyltransferase; Adds the first Dol-P-Man derived mannose in an alpha-1,3 linkage to Man5GlcNAc2-PP-Dol; Dolichyl D-mannosyl phosphate dependent mannosyltransferases
MAP2K1	Dual specificity mitogen-activated protein kinase kinase 1; Dual specificity protein kinase which acts as an essential component of the MAP kinase signal transduction pathway. Binding of extracellular ligands such as growth factors, cytokines and hormones to their cell-surface receptors activates RAS and this initiates RAF1 activation. RAF1 then further activates the dual-specificity protein kinases MAP2K1/MEK1 and MAP2K2/MEK2
RRAGC	Ras-related gtp-binding protein c/d; Ras-related GTP-binding protein C; Guanine nucleotide-binding protein forming heterodimeric Rag complexes required for the amino acid-induced relocalization of mTORC1 to the lysosomes and its subsequent activation by the GTPase RHEB. This is a crucial step in the activation of the TOR signaling cascade by amino acids
LAMTOR5	Ragulator complex protein LAMTOR5; As part of the Ragulator complex it is involved in amino acid sensing and activation of mTORC1, a signaling complex promoting cell growth in response to growth factors, energy levels, and amino acids. Activated by amino acids through a mechanism involving the lysosomal V-ATPase, the Ragulator functions as a guanine nucleotide exchange factor activating the small GTPases Rag. Activated Ragulator and Rag GTPases function as a scaffold recruiting mTORC1 to lysosomes where it is in turn activated
MAP2K2	Dual specificity mitogen-activated protein kinase kinase 2; Catalyzes the concomitant phosphorylation of a threonine and a tyrosine residue in a Thr-Glu-Tyr sequence located in MAP kinases. Activates the ERK1 and ERK2 MAP kinases (By similarity)
MECP2	Methyl-CpG-binding protein 2; Chromosomal protein that binds to methylated DNA. It can bind specifically to a single methyl-CpG pair. It is not influenced by sequences flanking the methyl-CpGs. Mediates transcriptional repression through interaction with histone deacetylase and the corepressor SIN3A. Binds both 5-methylcytosine (5mC) and 5-hydroxymethylcytosine (5hmC)-containing DNA, with a preference for 5-methylcytosine (5mC)
NPRL2	Nitrogen permease regulator 2-like protein; GATOR complex protein NPRL2; As a component of the GATOR1 complex functions as an inhibitor of the amino acid-sensing branch of the TORC1 pathway. The GATOR1 complex strongly increases GTP hydrolysis by RRAGA and RRAGB within RRAGC-containing heterodimers, thereby deactivating RRAGs, releasing mTORC1 from lysosomal surface and inhibiting mTORC1 signaling. The GATOR1 complex is negatively regulated by GATOR2 the other GATOR subcomplex in this amino acid-sensing branch of the TORC1 pathway; Belongs to the NPR2 family
DEPDC5	Dep domain containing 5, gator1 subcomplex subunit; GATOR complex protein DEPDC5; As a component of the GATOR1 complex functions as an inhibitor of the amino acid-sensing branch of the TORC1 pathway. The GATOR1 complex strongly increases GTP hydrolysis by RRAGA and RRAGB within RRAGC-containing heterodimers, thereby deactivating RRAGs, releasing mTORC1 from lysosomal surface and inhibiting mTORC1 signaling. The GATOR1 complex is negatively regulated by GATOR2 the other GATOR subcomplex in this amino acid-sensing branch of the TORC1 pathway
TSC1	Hamartin; In complex with TSC2, inhibits the nutrient-mediated or growth factor-stimulated phosphorylation of S6K1 and EIF4EBP1 by negatively regulating mTORC1 signaling. Seems not to be required for TSC2 GAP activity towards RHEB. Implicated as a tumor suppressor. Involved in microtubule-mediated protein transport, but this seems to be due to unregulated mTOR signaling; Armadillo-like helical domain containing
RHEB	GTP-binding protein Rheb; Activates the protein kinase activity of mTORC1, and thereby plays a role in the regulation of apoptosis. Stimulates the phosphorylation of S6K1 and EIF4EBP1 through activation of mTORC1 signaling. Has low intrinsic GTPase activity; RAS type GTPase family

LAMTOR1 Late endosomal/lysosomal adaptor, mapk and mtor activator 1; Ragulator complex protein LAMTOR1; As part of the Ragulator complex it is involved in amino acid sensing and activation of mTORC1, a signaling complex promoting cell growth in response to growth factors, energy levels, and amino acids. Activated by amino acids through a mechanism involving the lysosomal V-ATPase, the Ragulator functions as a guanine nucleotide exchange factor activating the small GTPases Rag

MTOR Serine/threonine-protein kinase mTOR; Serine/threonine protein kinase which is a central regulator of cellular metabolism, growth and survival in response to hormones, growth factors, nutrients, energy and stress signals. MTOR directly or indirectly regulates the phosphorylation of at least 800 proteins. Functions as part of 2 structurally and functionally distinct signaling complexes mTORC1 and mTORC2 (mTOR complex 1 and 2). Activated mTORC1 up-regulates protein synthesis by phosphorylating key regulators of mRNA translation and ribosome synthesis

SZT2 KICSTOR complex protein SZT2; As part of the KICSTOR complex functions in the amino acid-sensing branch of the TORC1 signaling pathway. Recruits, in an amino acid-independent manner, the GATOR1 complex to the lysosomal membranes and allows its interaction with GATOR2 and the RAG GTPases. Functions upstream of the RAG GTPases and is required to negatively regulate mTORC1 signaling in absence of amino acids. In absence of the KICSTOR complex mTORC1 is constitutively localized to the lysosome and activated

PAK3 Serine/threonine-protein kinase PAK 3; Serine/threonine protein kinase that plays a role in a variety of different signaling pathways including cytoskeleton regulation, cell migration, or cell cycle regulation. Plays a role in dendrite spine morphogenesis as well as synapse formation and plasticity. Acts as downstream effector of the small GTPases CDC42 and RAC1. Activation by the binding of active CDC42 and RAC1 results in a conformational change and a subsequent autophosphorylation on several serine and/or threonine residues

BRAF B-raf proto-oncogene serine/threonine-protein kinase; Serine/threonine-protein kinase B-raf; Protein kinase involved in the transduction of mitogenic signals from the cell membrane to the nucleus. May play a role in the postsynaptic responses of hippocampal neuron. Phosphorylates MAP2K1, and thereby contributes to the MAP kinase signal transduction pathway

RICTOR Rapamycin-insensitive companion of mTOR; Subunit of mTORC2, which regulates cell growth and survival in response to hormonal signals. mTORC2 is activated by growth factors, but, in contrast to mTORC1, seems to be nutrient- insensitive. mTORC2 seems to function upstream of Rho GTPases to regulate the actin cytoskeleton, probably by activating one or more Rho-type guanine nucleotide exchange factors. mTORC2 promotes the serum-induced formation of stress-fibers or F-actin

MLST8 Target of rapamycin complex subunit LST8; Subunit of both mTORC1 and mTORC2, which regulates cell growth and survival in response to nutrient and hormonal signals. mTORC1 is activated in response to growth factors or amino acids. Growth factor-stimulated mTORC1 activation involves a AKT1- mediated phosphorylation of TSC1-TSC2, which leads to the activation of the RHEB GTPase that potently activates the protein kinase activity of mTORC1. Amino acid-signaling to mTORC1 requires its relocalization to the lysosomes mediated by the Ragulator complex and the Rag GTPases.

PTEN Phosphatase and tensin homolog; Tumor suppressor. Acts as a dual-specificity protein phosphatase, dephosphorylating tyrosine-, serine- and threonine- phosphorylated proteins. Also acts as a lipid phosphatase, removing the phosphate in the D3 position of the inositol ring from phosphatidylinositol 3,4,5-trisphosphate, phosphatidylinositol 3,4-diphosphate, phosphatidylinositol 3- phosphate and inositol 1,3,4,5-tetrakisphosphate with order of substrate preference in vitro PtdIns(3,4,5)P3 > PtdIns(3,4)P2 > PtdIns3P > Ins(1,3,4,5)P4

IRS1 Insulin receptor substrate 1; May mediate the control of various cellular processes by insulin. When phosphorylated by the insulin receptor binds specifically to various cellular proteins containing SH2 domains such as phosphatidylinositol 3-kinase p85 subunit or GRB2. Activates phosphatidylinositol 3-kinase when bound to the regulatory p85 subunit (By similarity)

RAPTOR Regulatory-associated protein of mTOR; Involved in the control of the mammalian target of rapamycin complex 1 (mTORC1) activity which regulates cell growth and survival, and autophagy in response to nutrient and hormonal signals; functions as a scaffold for recruiting mTORC1 substrates. mTORC1 is activated in response to growth factors or amino acids. Growth factor-stimulated mTORC1 activation involves a AKT1- mediated phosphorylation of TSC1-TSC2, which leads to the activation of the RHEB GTPase that potently activates the protein kinase activity of mTORC1

PIK3CA Phosphatidylinositol 4,5-bisphosphate 3-kinase catalytic subunit alpha isoform; Phosphoinositide-3-kinase (PI3K) that phosphorylates PtdIns (Phosphatidylinositol), PtdIns4P (Phosphatidylinositol 4- phosphate) and PtdIns(4,5)P2 (Phosphatidylinositol 4,5- bisphosphate) to generate phosphatidylinositol 3,4,5-trisphosphate (PIP3). PIP3 plays a key role by recruiting

	PH domain-containing proteins to the membrane, including AKT1 and PDPK1, activating signaling cascades involved in cell growth, survival, proliferation, motility and morphology
TSC2	Tuberin; In complex with TSC1, this tumor suppressor inhibits the nutrient-mediated or growth factor-stimulated phosphorylation of S6K1 and EIF4EBP1 by negatively regulating mTORC1 signaling. Acts as a GTPase-activating protein (GAP) for the small GTPase RHEB, a direct activator of the protein kinase activity of mTORC1. May also play a role in microtubule-mediated protein transport. Also stimulates the intrinsic GTPase activity of the Ras-related proteins RAP1A and RAB5; Armadillo-like helical domain containing
KPTN	KICSTOR complex protein kaptin; As part of the KICSTOR complex functions in the amino acid-sensing branch of the TORC1 signaling pathway. Recruits, in an amino acid-independent manner, the GATOR1 complex to the lysosomal membranes and allows its interaction with GATOR2 and the RAG GTPases. Functions upstream of the RAG GTPases and is required to negatively regulate mTORC1 signaling in absence of amino acids. In absence of the KICSTOR complex mTORC1 is constitutively localized to the lysosome and activated. The KICSTOR complex is also probably involved in the regulation of mTORC1 by glucose
LAMTOR2	Ragulator complex protein LAMTOR2; As part of the Ragulator complex it is involved in amino acid sensing and activation of mTORC1, a signaling complex promoting cell growth in response to growth factors, energy levels, and amino acids. Activated by amino acids through a mechanism involving the lysosomal V-ATPase, the Ragulator functions as a guanine nucleotide exchange factor activating the small GTPases Rag. Activated Ragulator and Rag GTPases function as a scaffold recruiting mTORC1 to lysosomes where it is in turn activated
RRAGA	Ras-related gtp-binding protein a/b; Ras-related GTP-binding protein A; Guanine nucleotide-binding protein that plays a crucial role in the cellular response to amino acid availability through regulation of the mTORC1 signaling cascade. Forms heterodimeric Rag complexes with RRAGC or RRAGD and cycles between an inactive GDP-bound and an active GTP-bound form. In its active form participates in the relocalization of mTORC1 to the lysosomes and its subsequent activation by the GTPase RHEB. Involved in the RCC1/Ran-GTPase pathway
LAMTOR4	Ragulator complex protein LAMTOR4; As part of the Ragulator complex it is involved in amino acid sensing and activation of mTORC1, a signaling complex promoting cell growth in response to growth factors, energy levels, and amino acids. Activated by amino acids through a mechanism involving the lysosomal V-ATPase, the Ragulator functions as a guanine nucleotide exchange factor activating the small GTPases Rag. Activated Ragulator and Rag GTPases function as a scaffold recruiting mTORC1 to lysosomes where it is in turn activated
RPS6KA3	Ribosomal protein S6 kinase alpha-3; Serine/threonine-protein kinase that acts downstream of ERK (MAPK1/ERK2 and MAPK3/ERK1) signaling and mediates mitogenic and stress-induced activation of the transcription factors CREB1, ETV1/ER81 and NR4A1/NUR77, regulates translation through RPS6 and EIF4B phosphorylation, and mediates cellular proliferation, survival, and differentiation by modulating mTOR signaling and repressing pro-apoptotic function of BAD and DAPK1
NPRL3	Nitrogen permease regulator 3-like protein; GATOR complex protein NPRL3; As a component of the GATOR1 complex functions as an inhibitor of the amino acid-sensing branch of the TORC1 pathway. The GATOR1 complex strongly increases GTP hydrolysis by RRAGA and RRAGB within RRAGC-containing heterodimers, thereby deactivating RRAGs, releasing mTORC1 from lysosomal surface and inhibiting mTORC1 signaling. The GATOR1 complex is negatively regulated by GATOR2 the other GATOR subcomplex in this amino acid-sensing branch of the TORC1 pathway; Belongs to the NPR3 family
RPS6KB1	Ribosomal protein S6 kinase beta-1; Serine/threonine-protein kinase that acts downstream of mTOR signaling in response to growth factors and nutrients to promote cell proliferation, cell growth and cell cycle progression. Regulates protein synthesis through phosphorylation of EIF4B, RPS6 and EEF2K, and contributes to cell survival by repressing the pro-apoptotic function of BAD. Under conditions of nutrient depletion, the inactive form associates with the EIF3 translation initiation complex
AKT3	RAC-gamma serine/threonine-protein kinase; AKT3 is one of 3 closely related serine/threonine-protein kinases (AKT1, AKT2 and AKT3) called the AKT kinase, and which regulate many processes including metabolism, proliferation, cell survival, growth and angiogenesis. This is mediated through serine and/or threonine phosphorylation of a range of downstream substrates. Over 100 substrate candidates have been reported so far, but for most of them, no isoform specificity has been reported. AKT3 is the least studied AKT isoform. It plays an important role in brain development and is crucial f [...]
CDK19	Cyclin-dependent kinase 8/11; Cyclin dependent kinase 19; Belongs to the protein kinase superfamily. CMGC Ser/Thr protein kinase family. CDC2/CDKX subfamily
TCF4	Transcription factor 4; Transcription factor that binds to the immunoglobulin enchancer Mu-E5/KE5-motif. Involved in the initiation of neuronal differentiation. Activates transcription by

	binding to the E box (5'-CANNTG-3'). Binds to the E-box present in the somatostatin receptor 2 initiator element (SSTR2-INR) to activate transcription (By similarity). Preferentially binds to either 5'-ACANNTGT-3' or 5'-CCANNTGG-3'; Basic helix-loop-helix proteins
TAF13	Transcription initiation factor TFIID subunit 13; Component of the DNA-binding general RNA polymerase II transcription factor IID complex (TFIID). TFIID plays a critical role in the regulation of gene transcription in eukaryotic cells; Belongs to the TAF13 family
CREBBP	CREB-binding protein; Acetylates histones, giving a specific tag for transcriptional activation. Also acetylates non-histone proteins, like NCOA3 and FOXO1. Binds specifically to phosphorylated CREB and enhances its transcriptional activity toward cAMP-responsive genes. Acts as a coactivator of ALX1. Acts as a circadian transcriptional coactivator which enhances the activity of the circadian transcriptional activators: NPAS2-ARNTL/BMAL1 and CLOCK-ARNTL/BMAL1 heterodimers. Acetylates PCNA
ARS	Secreted Ly-6/uPAR-related protein 1; Has an antitumor activity. Was found to be a marker of late differentiation of the skin. Implicated in maintaining the physiological and structural integrity of the keratinocyte layers of the skin. In vitro down-regulates keratinocyte proliferation; the function may involve the proposed role as modulator of nicotinic acetylcholine receptors (nAChRs) activity. In vitro inhibits alpha-7-dependent nAChR currents in an allosteric manner. In T cells may be involved in regulation of intracellular Ca(2+) signaling
TAF10	Transcription initiation factor TFIID subunit 10; TAFs are components of the transcription factor IID (TFIID) complex, PCAF histone acetylase complex and TBP-free TAFII complex (TFTC). TIIFD is a multimeric protein complex that plays a central role in mediating promoter responses to various activators and repressors; Belongs to the TAF10 family
POLR3B	DNA-directed RNA polymerase III subunit RPC2; DNA-dependent RNA polymerase catalyzes the transcription of DNA into RNA using the four ribonucleoside triphosphates as substrates. Second largest core component of RNA polymerase III which synthesizes small RNAs, such as 5S rRNA and tRNAs. Proposed to contribute to the polymerase catalytic activity and forms the polymerase active center together with the largest subunit
TAF7	Transcription initiation factor TFIID subunit 7; Functions as a component of the DNA-binding general transcription factor complex TFIID, a multimeric protein complex that plays a central role in mediating promoter responses to various activators and repressors. Present in both of the previously described TFIID species which either lack or contain TAFII30 (TFIID alpha and TFIID beta respectively); Belongs to the TAF7 family
ATN1	Atrophin-1 (dentatorubral-pallidoluysian atrophy protein); Atrophin-1; Transcriptional corepressor. Recruits NR2E1 to repress transcription. Promotes vascular smooth cell (VSMC) migration and orientation (By similarity). Corepressor of MTG8 transcriptional repression. Has some intrinsic repression activity which is independent of the number of poly-Asn (polyQ) repeats
REST	RE1-silencing transcription factor; Transcriptional repressor which binds neuron-restrictive silencer element (NRSE) and represses neuronal gene transcription in non-neuronal cells. Restricts the expression of neuronal genes by associating with two distinct corepressors, mSin3 and CoREST, which in turn recruit histone deacetylase to the promoters of REST-regulated genes. Mediates repression by recruiting the BHC complex at RE1/NRSE sites which acts by deacetylating and demethylating specific sites on histones, thereby acting as a chromatin modifier
TAF1	Transcription initiation factor TFIID subunit 1; Largest component and core scaffold of the TFIID basal transcription factor complex. Contains novel N- and C-terminal Ser/Thr kinase domains which can autophosphorylate or transphosphorylate other transcription factors. Phosphorylates TP53 on 'Thr-55' which leads to MDM2- mediated degradation of TP53. Phosphorylates GTF2A1 and GTF2F1 on Ser residues. Possesses DNA-binding activity. Essential for progression of the G1 phase of the cell cycle. Exhibits histone acetyltransferase activity towards histones H3 and H4; Belongs to the TAF1 family
TAF11	Transcription initiation factor TFIID subunit 11; Core TAFII present in both of the previously described TFIID species which either lack or contain TAFII30 (TFIID alpha and TFIID beta respectively); Belongs to the TAF11 family
TAF8	Transcription initiation factor TFIID subunit 8; Transcription factor TFIID is one of the general factors required for accurate and regulated initiation by RNA polymerase II. Mediates both basal and activator-dependent transcription. Plays a role in the differentiation of preadipocyte fibroblasts to adipocytes, however, does not seem to play a role in differentiation of myoblasts. Required for the integration of TAF10 in the TAF complex. May be important for survival of cells of the inner cell mass which constitute the pluripotent cell population of the early embryo (By similarity)
MED17	Mediator of RNA polymerase II transcription subunit 17; Component of the Mediator complex, a coactivator involved in the regulated transcription of nearly all RNA polymerase II-dependent

	genes. Mediator functions as a bridge to convey information from gene-specific regulatory proteins to the basal RNA polymerase II transcription machinery. Mediator is recruited to promoters by direct interactions with regulatory proteins and serves as a scaffold for the assembly of a functional preinitiation complex with RNA polymerase II and the general transcription factors
POLR3A	DNA-directed RNA polymerase III subunit RPC1; DNA-dependent RNA polymerase catalyzes the transcription of DNA into RNA using the four ribonucleoside triphosphates as substrates. Largest and catalytic core component of RNA polymerase III which synthesizes small RNAs, such as 5S rRNA and tRNAs. Forms the polymerase active center together with the second largest subunit. A single-stranded DNA template strand of the promoter is positioned within the central active site cleft of Pol III
TAF11	Transcription initiation factor TFIID subunit 11; Core TAFII present in both of the previously described TFIID species which either lack or contain TAFII30 (TFIID alpha and TFIID beta respectively); Belongs to the TAF11 family
GTF2A1	Transcription initiation factor IIA subunit 1; TFIIA is a component of the transcription machinery of RNA polymerase II and plays an important role in transcriptional activation. TFIIA in a complex with TBP mediates transcriptional activity; Belongs to the TFIIA subunit 1 family
GTF2A2	Transcription initiation factor IIA subunit 2; TFIIA is a component of the transcription machinery of RNA polymerase II and plays an important role in transcriptional activation. TFIIA in a complex with TBP mediates transcriptional activity; General transcription factors
GTF2B	Transcription initiation factor IIB; General factor that plays a major role in the activation of eukaryotic genes transcribed by RNA polymerase II; Belongs to the TFIIB family
SMARCC1	Swi/snf related, matrix associated, actin dependent regulator of chromatin subfamily c member 1; SWI/SNF complex subunit SMARCC1; Involved in transcriptional activation and repression of select genes by chromatin remodeling (alteration of DNA-nucleosome topology). Component of SWI/SNF chromatin remodeling complexes that carry out key enzymatic activities, changing chromatin structure by altering DNA-histone contacts within a nucleosome in an ATP-dependent manner. May stimulate the ATPase activity of the catalytic subunit of the complex
SMARCC2	Swi/snf related, matrix associated, actin dependent regulator of chromatin subfamily c member 2; SWI/SNF complex subunit SMARCC2; Involved in transcriptional activation and repression of select genes by chromatin remodeling (alteration of DNA-nucleosome topology). Component of SWI/SNF chromatin remodeling complexes that carry out key enzymatic activities, changing chromatin structure by altering DNA-histone contacts within a nucleosome in an ATP-dependent manner. Can stimulate the ATPase activity of the catalytic subunit of these complexes
SMARCC2	Swi/snf related, matrix associated, actin dependent regulator of chromatin subfamily c member 2; SWI/SNF complex subunit SMARCC2; Involved in transcriptional activation and repression of select genes by chromatin remodeling (alteration of DNA-nucleosome topology). Component of SWI/SNF chromatin remodeling complexes that carry out key enzymatic activities, changing chromatin structure by altering DNA-histone contacts within a nucleosome in an ATP-dependent manner. Can stimulate the ATPase activity of the catalytic subunit of these complexes
SMARCB1	Swi/snf related, matrix associated, actin dependent regulator of chromatin, subfamily b, member 1; SWI/SNF-related matrix-associated actin-dependent regulator of chromatin subfamily B member 1; Core component of the BAF (hSWI/SNF) complex. This ATP- dependent chromatin-remodeling complex plays important roles in cell proliferation and differentiation, in cellular antiviral activities and inhibition of tumor formation. The BAF complex is able to create a stable, altered form of chromatin that constrains fewer negative supercoils than normal
SMARCA4	Swi/snf related, matrix associated, actin dependent regulator of chromatin, subfamily a, member 4; Transcription activator BRG1; Involved in transcriptional activation and repression of select genes by chromatin remodeling (alteration of DNA-nucleosome topology). Component of SWI/SNF chromatin remodeling complexes that carry out key enzymatic activities, changing chromatin structure by altering DNA-histone contacts within a nucleosome in an ATP-dependent manner
ACTL6B	Actin-like protein 6B; Involved in transcriptional activation and repression of select genes by chromatin remodeling (alteration of DNA-nucleosome topology). Component of SWI/SNF chromatin remodeling complexes that carry out key enzymatic activities, changing chromatin structure by altering DNA-histone contacts within a nucleosome in an ATP-dependent manner.
WDR5	WD repeat-containing protein 5; Contributes to histone modification. May position the N-terminus of histone H3 for efficient trimethylation at 'Lys-4'. As part of the MLL1/MLL complex it is involved in methylation and dimethylation at 'Lys-4' of histone H3. H3 'Lys-4' methylation represents a specific tag for epigenetic transcriptional activation. As part of the

	NSL complex it may be involved in acetylation of nucleosomal histone H4 on several lysine residues. May regulate osteoblasts differentiation; Belongs to the WD repeat WDR5/wds family
KDM5C	Lysine-specific demethylase 5C; Histone demethylase that specifically demethylates 'Lys- 4' of histone H3, thereby playing a central role in histone code. Does not demethylate histone H3 'Lys-9', H3 'Lys-27', H3 'Lys-36', H3 'Lys-79' or H4 'Lys-20'. Demethylates trimethylated and dimethylated but not monomethylated H3 'Lys-4'. Participates in transcriptional repression of neuronal genes by recruiting histone deacetylases and REST at neuron-restrictive silencer elements
ATRX	Transcriptional regulator ATRX; Involved in transcriptional regulation and chromatin remodeling. Facilitates DNA replication in multiple cellular environments and is required for efficient replication of a subset of genomic loci. Binds to DNA tandem repeat sequences in both telomeres and euchromatin and in vitro binds DNA quadruplex structures. May help stabilizing G-rich regions into regular chromatin structures by remodeling G4 DNA and incorporating H3.3- containing nucleosomes
EIF2B1	Translation initiation factor eIF-2B subunit alpha; Catalyzes the exchange of eukaryotic initiation factor 2-bound GDP for GTP
EIF2B5	Translation initiation factor eIF-2B subunit epsilon; Catalyzes the exchange of eukaryotic initiation factor 2-bound GDP for GTP; Belongs to the eIF-2B gamma/epsilon subunits family
EIF2B4	Translation initiation factor eIF-2B subunit delta; Catalyzes the exchange of eukaryotic initiation factor 2-bound GDP for GTP
EIF2B2	Translation initiation factor eIF-2B subunit beta; Catalyzes the exchange of eukaryotic initiation factor 2-bound GDP for GTP; Belongs to the eIF-2B alpha/beta/delta subunits family
EIF2B3	Translation initiation factor eIF-2B subunit gamma; Catalyzes the exchange of eukaryotic initiation factor 2-bound GDP for GTP; Belongs to the eIF-2B gamma/epsilon subunits family
EIF2S1	Eukaryotic translation initiation factor 2 subunit 1; Functions in the early steps of protein synthesis by forming a ternary complex with GTP and initiator tRNA. This complex binds to a 40S ribosomal subunit, followed by mRNA binding to form a 43S pre-initiation complex. Junction of the 60S ribosomal subunit to form the 80S initiation complex is preceded by hydrolysis of the GTP bound to eIF-2 and release of an eIF-2- GDP binary complex
AIMP1	Aminoacyl tRNA synthase complex-interacting multifunctional protein 1; Non-catalytic component of the multisynthase complex. Stimulates the catalytic activity of cytoplasmic arginyl-tRNA synthase. Binds tRNA. Possesses inflammatory cytokine activity. Negatively regulates TGF-beta signaling through stabilization of SMURF2 by binding to SMURF2 and inhibiting its SMAD7-mediated degradation. Involved in glucose homeostasis through induction of glucagon secretion at low glucose levels. Promotes dermal fibroblast proliferation and wound repair
DARS	Aspartate--tRNA ligase, cytoplasmic; Catalyzes the specific attachment of an amino acid to its cognate tRNA in a 2 step reaction: the amino acid (AA) is first activated by ATP to form AA-AMP and then transferred to the acceptor end of the tRNA; Belongs to the class-II aminoacyl-tRNA synthetase family. Type 2 subfamily
EPRS	Bifunctional glutamate/proline--tRNA ligase; Catalyzes the attachment of the cognate amino acid to the corresponding tRNA in a two-step reaction: the amino acid is first activated by ATP to form a covalent intermediate with AMP and is then transferred to the acceptor end of the cognate tRNA. Component of the GAIT (gamma interferon-activated inhibitor of translation) complex which mediates interferon-gamma-induced transcript- selective translation inhibition in inflammation processes.
RARS2	Probable arginine--tRNA ligase, mitochondrial; arginyl-tRNA synthetase 2, mitochondrial; Aminoacyl tRNA synthetases, Class I
AARS	Alanine--tRNA ligase, cytoplasmic; Catalyzes the attachment of alanine to tRNA(Ala) in a two-step reaction: alanine is first activated by ATP to form Ala- AMP and then transferred to the acceptor end of tRNA(Ala). Also edits incorrectly charged tRNA(Ala) via its editing domain; Belongs to the class-II aminoacyl-tRNA synthetase family
EEF1A2	Elongation factor 1-alpha 2; This protein promotes the GTP-dependent binding of aminoacyl-tRNA to the A-site of ribosomes during protein biosynthesis; Belongs to the TRAFAC class translation factor GTPase superfamily. Classic translation factor GTPase family. EF-Tu/EF-1A subfamily
GRIN1	Glutamate receptor ionotropic, NMDA 1; Component of NMDA receptor complexes that function as heterotetrameric, ligand-gated ion channels with high calcium permeability and voltage-dependent sensitivity to magnesium. Channel activation requires binding of the neurotransmitter glutamate to the epsilon subunit, glycine binding to the zeta subunit, plus membrane depolarization to eliminate channel inhibition by Mg(2+). Sensitivity to glutamate and channel kinetics depend on the subunit composition; Belongs to the glutamate-gated ion channel (TC 1.A.10.1) family. NR1/GRIN1 subfamily

GRIN2A	Glutamate receptor ionotropic, NMDA 2A; Component of NMDA receptor complexes that function as heterotetrameric, ligand-gated ion channels with high calcium permeability and voltage-dependent sensitivity to magnesium. Channel activation requires binding of the neurotransmitter glutamate to the epsilon subunit, glycine binding to the zeta subunit, plus membrane depolarization to eliminate channel inhibition by Mg(2+).
SYNGAP1	Ras/Rap GTPase-activating protein SynGAP; Major constituent of the PSD essential for postsynaptic signaling. Inhibitory regulator of the Ras-cAMP pathway. Member of the NMDAR signaling complex in excitatory synapses, it may play a role in NMDAR-dependent control of AMPAR potentiation, AMPAR membrane trafficking and synaptic plasticity. Regulates AMPAR- mediated miniature excitatory postsynaptic currents. Exhibits dual GTPase-activating specificity for Ras and Rap.
DLG3	Disks large homolog 3; Required for learning most likely through its role in synaptic plasticity following NMDA receptor signaling; Membrane associated guanylate kinases
GRIN2B	Glutamate receptor ionotropic, NMDA 2B; Component of NMDA receptor complexes that function as heterotetrameric, ligand-gated ion channels with high calcium permeability and voltage-dependent sensitivity to magnesium. Channel activation requires binding of the neurotransmitter glutamate to the epsilon subunit, glycine binding to the zeta subunit, plus membrane depolarization to eliminate channel inhibition by Mg(2+). Sensitivity to glutamate and channel kinetics depend on the subunit composition.
KCNJ10	ATP-sensitive inward rectifier potassium channel 10; May be responsible for potassium buffering action of glial cells in the brain. Inward rectifier potassium channels are characterized by a greater tendency to allow potassium to flow into the cell rather than out of it. Their voltage dependence is regulated by the concentration of extracellular potassium; as external potassium is raised, the voltage range of the channel opening shifts to more positive voltages. The inward rectification is mainly due to the blockage of outward current by internal magnesium.
GNAO1	Guanine nucleotide-binding protein G(o) subunit alpha; Guanine nucleotide-binding proteins (G proteins) are involved as modulators or transducers in various transmembrane signaling systems. The G(o) protein function is not clear. Stimulated by RGS14; Belongs to the G-alpha family. G(i/o/t/z) subfamily
GRIA3	Glutamate receptor 3; Receptor for glutamate that functions as ligand-gated ion channel in the central nervous system and plays an important role in excitatory synaptic transmission. L-glutamate acts as an excitatory neurotransmitter at many synapses in the central nervous system. Binding of the excitatory neurotransmitter L- glutamate induces a conformation change, leading to the opening of the cation channel, and thereby converts the chemical signal to an electrical impulse.
ADAM22	Disintegrin and metalloproteinase domain-containing protein 22; Probable ligand for integrin in the brain. This is a non catalytic metalloprotease-like protein. Involved in regulation of cell adhesion and spreading and in inhibition of cell proliferation. Neuronal receptor for LGI1; ADAM metallopeptidase domain containing
DLG4	Disks large homolog 4; Interacts with the cytoplasmic tail of NMDA receptor subunits and shaker-type potassium channels. Required for synaptic plasticity associated with NMDA receptor signaling. Overexpression or depletion of DLG4 changes the ratio of excitatory to inhibitory synapses in hippocampal neurons. May reduce the amplitude of ASIC3 acid-evoked currents by retaining the channel intracellularly. May regulate the intracellular trafficking of ADR1B (By similarity); Belongs to the MAGUK family
LGI1	Leucine-rich glioma-inactivated protein 1; Regulates voltage-gated potassium channels assembled from KCNA1, KCNA4 and KCNAB1. It slows down channel inactivation by precluding channel closure mediated by the KCNAB1 subunit. Ligand for ADAM22 that positively regulates synaptic transmission mediated by AMPA-type glutamate receptors (By similarity). Plays a role in suppressing the production of MMP1/3 through the phosphatidylinositol 3-kinase/ERK pathway. May play a role in the control of neuroblastoma cell survival
TUBA1A	Tubulin alpha-1A chain; Tubulin is the major constituent of microtubules. It binds two moles of GTP, one at an exchangeable site on the beta chain and one at a non-exchangeable site on the alpha chain; Tubulins
RNASEH2B	Ribonuclease H2 subunit B; Non catalytic subunit of RNase H2, an endonuclease that specifically degrades the RNA of RNA:DNA hybrids. Participates in DNA replication, possibly by mediating the removal of lagging- strand Okazaki fragment RNA primers during DNA replication. Mediates the excision of single ribonucleotides from DNA:RNA duplexes
STILL	Mitogen-activated protein kinase 6; Atypical MAPK protein. Phosphorylates microtubule-associated protein 2 (MAP2) and MAPKAPK5. The precise role of the complex formed with MAPKAPK5 is still unclear, but the complex follows a complex set of phosphorylation events: upon interaction with atypical MAPKAPK5, ERK3/MAPK6 is phosphorylated at Ser-189 and

	then mediates phosphorylation and activation of MAPKAPK5, which in turn phosphorylates ERK3/MAPK6. May promote entry in the cell cycle (By similarity); Mitogen-activated protein kinases
SMC1A	Structural maintenance of chromosomes protein 1A; Involved in chromosome cohesion during cell cycle and in DNA repair. Central component of cohesin complex. The cohesin complex is required for the cohesion of sister chromatids after DNA replication. The cohesin complex apparently forms a large proteinaceous ring within which sister chromatids can be trapped. At anaphase, the complex is cleaved and dissociates from chromatin, allowing sister chromatids to segregate.
RNASEH2A	Ribonuclease H2 subunit A; Catalytic subunit of RNase HII, an endonuclease that specifically degrades the RNA of RNA:DNA hybrids. Participates in DNA replication, possibly by mediating the removal of lagging- strand Okazaki fragment RNA primers during DNA replication. Mediates the excision of single ribonucleotides from DNA:RNA duplexes; Belongs to the RNase HII family. Eukaryotic subfamily
TUBB2B	Tubulin beta-2B chain; Tubulin is the major constituent of microtubules. It binds two moles of GTP, one at an exchangeable site on the beta chain and one at a non-exchangeable site on the alpha chain (By similarity). Plays a critical role in proper axon guidance in both central and peripheral axon tracts. Implicated in neuronal migration; Tubulins
KIF7	Kinesin-like protein KIF7; Essential for hedgehog signaling regulation: acts as both a negative and positive regulator of sonic hedgehog (Shh) and Indian hedgehog (Ihh) pathways, acting downstream of SMO, through both SUFU-dependent and -independent mechanisms. Involved in the regulation of microtubular dynamics. Required for proper organization of the ciliary tip and control of ciliary localization of SUFU-GLI2 complexes (By similarity). Required for localization of GLI3 to cilia in response to Shh.
RNASEH2C	Ribonuclease H2 subunit C; Non catalytic subunit of RNase H2, an endonuclease that specifically degrades the RNA of RNA:DNA hybrids. Participates in DNA replication, possibly by mediating the removal of lagging- strand Okazaki fragment RNA primers during DNA replication. Mediates the excision of single ribonucleotides from DNA:RNA duplexes
ATR	Serine/threonine-protein kinase ATR; Serine/threonine protein kinase which activates checkpoint signaling upon genotoxic stresses such as ionizing radiation (IR), ultraviolet light (UV), or DNA replication stalling, thereby acting as a DNA damage sensor. Recognizes the substrate consensus sequence [ST]-Q. Phosphorylates BRCA1, CHEK1, MCM2, RAD17, RPA2, SMC1 and p53/TP53, which collectively inhibit DNA replication and mitosis and promote DNA repair, recombination and apoptosis.
NIPBL	Cohesin loading factor subunit scc2; Nipped-B-like protein; Along with ZNF609, promotes cortical neuron migration during brain development by regulating the transcription of crucial genes in this process. Preferentially binds promoters containing paused RNA polymerase II. Up-regulates the expression of SEMA3A, NRP1, PLXND1 and GABBR2 genes, among others; Armadillo-like helical domain containing
TUBB2A	Tubulin beta-2A chain; Tubulin is the major constituent of microtubules. It binds two moles of GTP, one at an exchangeable site on the beta chain and one at a non-exchangeable site on the alpha chain (By similarity); Tubulins
FGF13	Fibroblast growth factor 13; Microtubule-binding protein which directly binds tubulin and is involved in both polymerization and stabilization of microtubules. Through its action on microtubules, may participate to the refinement of axons by negatively regulating axonal and leading processes branching. Plays a crucial role in neuron polarization and migration in the cerebral cortex and the hippocampus; Belongs to the heparin-binding growth factors family
CEP135	Centrosomal protein of 135 kDa; Centrosomal protein involved in centriole biogenesis. Acts as a scaffolding protein during early centriole biogenesis. Required for the targeting of centriole satellite proteins to centrosomes such as of PCM1, SSX2IP and CEP290 and recruitment of WRAP73 to centrioles. Also required for centriole-centriole cohesion during interphase by acting as a platform protein for CEP250 at the centriole; Belongs to the CEP135/TSGA10 family
CEP152	Centrosomal protein of 152 kDa; Necessary for centrosome duplication; the function seems also to involve CEP63, CDK5RAP2 and WDR62 through a stepwise assembled complex at the centrosome that recruits CDK2 required for centriole duplication. Acts as a molecular scaffold facilitating the interaction of PLK4 and CENPJ, 2 molecules involved in centriole formation. Proposed to snatch PLK4 away from PLK4:CEP92 complexes in early G1 daughter centriole and to reposition PLK4 at the outer boundary of a newly forming CEP152 ring structure
BUB1B	Mitotic checkpoint serine/threonine-protein kinase BUB1 beta; Essential component of the mitotic checkpoint. Required for normal mitosis progression. The mitotic checkpoint delays anaphase until all chromosomes are properly attached to the mitotic spindle. One of its checkpoint functions is to inhibit the activity of the anaphase-promoting complex/cyclosome

	(APC/C) by blocking the binding of CDC20 to APC/C, independently of its kinase activity. The other is to monitor kinetochore activities that depend on the kinetochore motor CENPE. Required for kinetochore localization of CENPE
PAFAH1B1	Platelet-activating factor acetylhydrolase IB subunit alpha; Required for proper activation of Rho GTPases and actin polymerization at the leading edge of locomoting cerebellar neurons and postmigratory hippocampal neurons in response to calcium influx triggered via NMDA receptors. Non-catalytic subunit of an acetylhydrolase complex which inactivates platelet-activating factor (PAF) by removing the acetyl group at the SN-2 position (By similarity). Positively regulates the activity of the minus-end directed microtubule motor protein dynein
SASS6	Spindle assembly abnormal protein 6 homolog; Central scaffolding component of the centrioles ensuring their 9-fold symmetry. Required for centrosome biogenesis and duplication: required both for mother-centriole-dependent centriole duplication and deuterosome-dependent centriole amplification in multiciliated cells. Overexpression results in excess foci-bearing centriolar markers. Required for the recruitment of STIL to the procentriole and for STIL-mediated centriole amplification
CSPP1	Centrosome and spindle pole-associated protein 1; May play a role in cell-cycle-dependent microtubule organization
CASC5	Kinetochore scaffold 1; Performs two crucial functions during mitosis: it is essential for spindle-assembly checkpoint signaling and for correct chromosome alignment. Required for attachment of the kinetochores to the spindle microtubules. Directly links BUB1 and BUB1B to kinetochores. Part of the MIS12 complex, which may be fundamental for kinetochore formation and proper chromosome segregation during mitosis. Acts in coordination with CENPK to recruit the NDC80 complex to the outer kinetochore
SMC3	Structural maintenance of chromosome 3 (chondroitin sulfate proteoglycan 6); Structural maintenance of chromosomes protein 3; Central component of cohesin, a complex required for chromosome cohesion during the cell cycle. The cohesin complex may form a large proteinaceous ring within which sister chromatids can be trapped. At anaphase, the complex is cleaved and dissociates from chromatin, allowing sister chromatids to segregate. Cohesion is coupled to DNA replication and is involved in DNA repair.
TUBA8	Tubulin alpha-8 chain; Tubulin is the major constituent of microtubules. It binds two moles of GTP, one at an exchangeable site on the beta chain and one at a non-exchangeable site on the alpha chain; Tubulins
ASPM	Abnormal spindle-like microcephaly-associated protein; Involved in mitotic spindle regulation and coordination of mitotic processes. The function in regulating microtubule dynamics at spindle poles including spindle orientation, astral microtubule density and poleward microtubule flux seems to depend on the association with the katanin complex formed by KATNA1 and KATNB1. Enhances the microtubule lattice severing activity of KATNA1 by recruiting the katanin complex to microtubules.
CEP290	Centrosomal protein of 290 kDa; Involved in early and late steps in cilia formation. Its association with CCP110 is required for inhibition of primary cilia formation by CCP110. May play a role in early ciliogenesis in the disappearance of centriolar satellites and in the transition of primary ciliar vesicles (PCVs) to capped ciliary vesicles (CCVs). Required for the centrosomal recruitment of RAB8A and for the targeting of centriole satellite proteins to centrosomes such as of PCM1.
EZH2	Histone-lysine N-methyltransferase EZH2; Polycomb group (PcG) protein. Catalytic subunit of the PRC2/EED-EZH2 complex, which methylates 'Lys-9' (H3K9me) and 'Lys- 27' (H3K27me) of histone H3, leading to transcriptional repression of the affected target gene. Able to mono-, di- and trimethylate 'Lys-27' of histone H3 to form H3K27me1, H3K27me2 and H3K27me3, respectively. Displays a preference for substrates with less methylation, loses activity when progressively more methyl groups are incorporated into H3K27, H3K27me0 > H3K27me1 > H3K27me2.
CEP63	Centrosomal protein of 63 kDa; Required for normal spindle assembly. Plays a key role in mother-centriole-dependent centriole duplication; the function seems also to involve CEP152, CDK5RAP2 and WDR62 through a stepwise assembled complex at the centrosome that recruits CDK2 required for centriole duplication. Reported to be required for centrosomal recruitment of CEP152; however, this function has been questioned. Also recruits CDK1 to centrosomes.
TUBB	Tubulin beta chain; Tubulin is the major constituent of microtubules. It binds two moles of GTP, one at an exchangeable site on the beta chain and one at a non-exchangeable site on the alpha chain; Tubulins
KIF2A	Kinesin-like protein KIF2A; Plus end-directed microtubule-dependent motor required for normal brain development. May regulate microtubule dynamics during axonal growth. Required for normal progression through mitosis. Required for normal congress of chromosomes at the metaphase plate. Required for normal spindle dynamics during mitosis. Promotes spindle

	turnover. Implicated in formation of bipolar mitotic spindles. Has microtubule depolymerization activity; Kinesins
ACTG1	Actin, cytoplasmic 2; Actins are highly conserved proteins that are involved in various types of cell motility and are ubiquitously expressed in all eukaryotic cells; Belongs to the actin family
TUBG1	Tubulin gamma-1 chain; Tubulin is the major constituent of microtubules. The gamma chain is found at microtubule organizing centers (MTOC) such as the spindle poles or the centrosome. Pericentriolar matrix component that regulates alpha/beta chain minus-end nucleation, centrosome duplication and spindle formation; Tubulins
DCX	Neuronal migration protein doublecortin; Microtubule-associated protein required for initial steps of neuronal dispersion and cortex lamination during cerebral cortex development. May act by competing with the putative neuronal protein kinase DCLK1 in binding to a target protein. May in that way participate in a signaling pathway that is crucial for neuronal interaction before and during migration, possibly as part of a calcium ion-dependent signal transduction pathway. May be part with PAFAH1B1/LIS-1 of overlapping, but distinct, signaling pathways that promote neuronal migration
CENPE	Centromere-associated protein E; Microtubule plus-end-directed kinetochore motor which plays an important role in chromosome congression, microtubule- kinetochore conjugation and spindle assembly checkpoint activation. Drives chromosome congression (alignment of chromosomes at the spindle equator resulting in the formation of the metaphase plate) by mediating the lateral sliding of polar chromosomes along spindle microtubules towards the spindle equator and by aiding the establishment and maintenance of connections between kinetochores and spindle microtubules.
MCPH1	Microcephalin 1; Microcephalin; Implicated in chromosome condensation and DNA damage induced cellular responses. May play a role in neurogenesis and regulation of the size of the cerebral cortex
OFD1	Oral-facial-digital syndrome 1 protein; Component of the centrioles controlling mother and daughter centrioles length. Recruits to the centriole IFT88 and centriole distal appendage-specific proteins including CEP164. Involved in the biogenesis of the cilium, a centriole-associated function. The cilium is a cell surface projection found in many vertebrate cells required to transduce signals important for development and tissue homeostasis. Plays an important role in development by regulating Wnt signaling and the specification of the left-right axis.
TUBB3	Tubulin beta-3 chain; Tubulin is the major constituent of microtubules. It binds two moles of GTP, one at an exchangeable site on the beta chain and one at a non-exchangeable site on the alpha chain. TUBB3 plays a critical role in proper axon guidance and mantainance; Belongs to the tubulin family
CDK5RAP2	CDK5 regulatory subunit-associated protein 2; Potential regulator of CDK5 activity via its interaction with CDK5R1. Negative regulator of centriole disengagement (licensing) which maintains centriole engagement and cohesion. Involved in regulation of mitotic spindle orientation (By similarity). Plays a role in the spindle checkpoint activation by acting as a transcriptional regulator of both BUBR1 and MAD2 promoter. Together with MAPRE1, it may promote microtubule polymerization, bundle formation, growth and dynamics at the plus ends.
ANAPC4	Anaphase-promoting complex subunit 4; Component of the anaphase promoting complex/cyclosome (APC/C), a cell cycle-regulated E3 ubiquitin ligase that controls progression through mitosis and the G1 phase of the cell cycle. The APC/C complex acts by mediating ubiquitination and subsequent degradation of target proteins: it mainly mediates the formation of 'Lys-11'-linked polyubiquitin chains and, to a lower extent, the formation of 'Lys-48'- and 'Lys-63'-linked polyubiquitin chains
HDAC8	Histone deacetylase 8; Responsible for the deacetylation of lysine residues on the N-terminal part of the core histones (H2A, H2B, H3 and H4). Histone deacetylation gives a tag for epigenetic repression and plays an important role in transcriptional regulation, cell cycle progression and developmental events. Histone deacetylases act via the formation of large multiprotein complexes. Also involved in the deacetylation of cohesin complex protein SMC3 regulating release of cohesin complexes from chromatin. May play a role in smooth muscle cell contractility
PCNT	Pericentrin; Integral component of the filamentous matrix of the centrosome involved in the initial establishment of organized microtubule arrays in both mitosis and meiosis. Plays a role, together with DISC1, in the microtubule network formation. Is an integral component of the pericentriolar material (PCM). May play an important role in preventing premature centrosome splitting during interphase by inhibiting NEK2 kinase activity at the centrosome
CENPJ	Centromere protein J; Plays an important role in cell division and centrosome function by participating in centriole duplication. Inhibits microtubule nucleation from the centrosome. Involved in the regulation of slow processive growth of centriolar microtubules. Acts as

	microtubule plus-end tracking protein that stabilizes centriolar microtubules and inhibits microtubule polymerization and extension from the distal ends of centrioles. Required for centriole elongation and for STIL- mediated centriole amplification.
MAD2L1	Mitotic spindle assembly checkpoint protein MAD2A; Component of the spindle-assembly checkpoint that prevents the onset of anaphase until all chromosomes are properly aligned at the metaphase plate. Required for the execution of the mitotic checkpoint which monitors the process of kinetochore- spindle attachment and inhibits the activity of the anaphase promoting complex by sequestering CDC20 until all chromosomes are aligned at the metaphase plate
KIF5C	Kinesin heavy chain isoform 5C; Mediates dendritic trafficking of mRNAs (By similarity). Kinesin is a microtubule-associated force-producing protein that may play a role in organelle transport; Belongs to the TRAFAC class myosin-kinesin ATPase superfamily. Kinesin family. Kinesin subfamily
NDE1	Nuclear distribution protein nudE homolog 1; Required for centrosome duplication and formation and function of the mitotic spindle. Essential for the development of the cerebral cortex. May regulate the production of neurons by controlling the orientation of the mitotic spindle during division of cortical neuronal progenitors of the proliferative ventricular zone of the brain.
RAD21	Double-strand-break repair protein rad21 homolog; Cleavable component of the cohesin complex, involved in chromosome cohesion during cell cycle, in DNA repair, and in apoptosis. The cohesin complex is required for the cohesion of sister chromatids after DNA replication. The cohesin complex apparently forms a large proteinaceous ring within which sister chromatids can be trapped. At metaphase-anaphase transition, this protein is cleaved by separase/ESPL1 and dissociates from chromatin, allowing sister chromatids to segregate.
CDC20	Cell division cycle protein 20 homolog; Required for full ubiquitin ligase activity of the anaphase promoting complex/cyclosome (APC/C) and may confer substrate specificity upon the complex. Is regulated by MAD2L1: in metaphase the MAD2L1-CDC20-APC/C ternary complex is inactive and in anaphase the CDC20-APC/C binary complex is active in degrading substrates.
RRM2B	Ribonucleotide reductase regulatory tp53 inducible subunit m2b; Ribonucleoside-diphosphate reductase subunit M2 B; Plays a pivotal role in cell survival by repairing damaged DNA in a p53/TP53-dependent manner. Supplies deoxyribonucleotides for DNA repair in cells arrested at G1 or G2. Contains an iron-tyrosyl free radical center required for catalysis. Forms an active ribonucleotide reductase (RNR) complex with RRM1 which is expressed both in resting and proliferating cells in response to DNA damage
DYNC1H1	Cytoplasmic dynein 1 heavy chain 1; Cytoplasmic dynein 1 acts as a motor for the intracellular retrograde motility of vesicles and organelles along microtubules. Dynein has ATPase activity; the force-producing power stroke is thought to occur on release of ADP. Plays a role in mitotic spindle assembly and metaphase plate congression; Belongs to the dynein heavy chain family
STAG1	Cohesin subunit SA-1; Component of cohesin complex, a complex required for the cohesion of sister chromatids after DNA replication. The cohesin complex apparently forms a large proteinaceous ring within which sister chromatids can be trapped. At anaphase, the complex is cleaved and dissociates from chromatin, allowing sister chromatids to segregate. The cohesin complex may also play a role in spindle pole assembly during mitosis; Armadillo-like helical domain containing
STAG2	Cohesin subunit SA-2; Component of cohesin complex, a complex required for the cohesion of sister chromatids after DNA replication. The cohesin complex apparently forms a large proteinaceous ring within which sister chromatids can be trapped. At anaphase, the complex is cleaved and dissociates from chromatin, allowing sister chromatids to segregate. The cohesin complex may also play a role in spindle pole assembly during mitosis; Belongs to the SCC3 family
PEX5	Peroxisomal targeting signal 1 receptor; Binds to the C-terminal PTS1-type tripeptide peroxisomal targeting signal (SKL-type) and plays an essential role in peroxisomal protein import
PEX2	Peroxisome biogenesis factor 2; Somewhat implicated in the biogenesis of peroxisomes; Belongs to the pex2/pex10/pex12 family
PEX7	Peroxisomal targeting signal 2 receptor; Binds to the N-terminal PTS2-type peroxisomal targeting signal and plays an essential role in peroxisomal protein import; Belongs to the WD repeat peroxin-7 family
PEX6	Peroxisomal biogenesis factor 6; Peroxisome assembly factor 2; Involved in peroxisome biosynthesis. Required for stability of the PTS1 receptor. Anchored by PEX26 to peroxisome membranes, possibly to form heteromeric AAA ATPase complexes required for the import of proteins into peroxisomes

PEX3	Peroxisomal biogenesis factor 3; Involved in peroxisome biosynthesis and integrity. Assembles membrane vesicles before the matrix proteins are translocated. As a docking factor for PEX19, is necessary for the import of peroxisomal membrane proteins in the peroxisomes; Belongs to the peroxin-3 family
PEX12	Peroxisomal biogenesis factor 12; Peroxisome assembly protein 12; Required for protein import into peroxisomes; Peroxins
ABCD1	ATP-binding cassette sub-family D member 1; Probable transporter. The nucleotide-binding fold acts as an ATP-binding subunit with ATPase activity; Belongs to the ABC transporter superfamily. ABCD family. Peroxisomal fatty acyl CoA transporter (TC 3.A.1.203) subfamily
PEX16	Peroxisomal membrane protein PEX16; Required for peroxisome membrane biogenesis. May play a role in early stages of peroxisome assembly. Can recruit other peroxisomal proteins, such as PEX3 and PMP34, to de novo peroxisomes derived from the endoplasmic reticulum (ER). May function as receptor for PEX3; Belongs to the peroxin-16 family
PEX14	Peroxisomal membrane protein PEX14; Peroxisome membrane protein that is an essential component of the peroxisomal import machinery. Functions as a docking factor for the predominantly cytoplasmic PTS1 receptor (PEX5). Plays a key role for peroxisome movement through a direct interaction with tubulin; Peroxins
PEX26	Peroxisomal biogenesis factor 26; Peroxisome assembly protein 26; Probably required for protein import into peroxisomes. Anchors PEX1 and PEX6 to peroxisome membranes, possibly to form heteromeric AAA ATPase complexes required for the import of proteins into peroxisomes. Involved in the import of catalase and proteins containing a PTS2 target sequence, but not in import of proteins with a PTS1 target sequence; Peroxins
PEX19	Peroxisomal biogenesis factor 19; Necessary for early peroxisomal biogenesis. Acts both as a cytosolic chaperone and as an import receptor for peroxisomal membrane proteins (PMPs). Binds and stabilizes newly synthesized PMPs in the cytoplasm by interacting with their hydrophobic membrane-spanning domains, and targets them to the peroxisome membrane by binding to the integral membrane protein PEX3. Excludes CDKN2A from the nucleus and prevents its interaction with MDM2, which results in active degradation of TP53; Belongs to the peroxin-19 family
PEX11B	Peroxisomal membrane protein 11B; Involved in peroxisomal proliferation. May regulate peroxisome division by recruiting the dynamin-related GTPase DNM1L to the peroxisomal membrane. Promotes membrane protrusion and elongation on the peroxisomal surface; Belongs to the peroxin-11 family
PEX13	Peroxisomal membrane protein PEX13; Component of the peroxisomal translocation machinery with PEX14 and PEX17. Functions as a docking factor for the predominantly cytoplasmic PTS1 receptor (PAS10/PEX5). Involved in the import of PTS1 and PTS2 proteins; Peroxins
PEX1	Peroxisomal biogenesis factor 1; Peroxisome biogenesis factor 1; Required for stability of PEX5 and protein import into the peroxisome matrix. Anchored by PEX26 to peroxisome membranes, possibly to form heteromeric AAA ATPase complexes required for the import of proteins into peroxisomes
GABRA1	Gamma-aminobutyric acid receptor subunit alpha-1; Component of the heteropentameric receptor for GABA, the major inhibitory neurotransmitter in the vertebrate brain. Functions also as histamine receptor and mediates cellular responses to histamine. Functions as receptor for diazepines and various anesthetics, such as pentobarbital; these are bound at a separate allosteric effector binding site. Functions as ligand- gated chloride channel (By similarity); Gamma-aminobutyric acid type A receptor subunits
GABRA2	Gamma-aminobutyric acid receptor subunit alpha-2; GABA, the major inhibitory neurotransmitter in the vertebrate brain, mediates neuronal inhibition by binding to the GABA/benzodiazepine receptor and opening an integral chloride channel; Gamma-aminobutyric acid type A receptor subunits
GABRG2	Gamma-aminobutyric acid receptor subunit gamma-2; Component of the heteropentameric receptor for GABA, the major inhibitory neurotransmitter in the vertebrate brain. Functions also as histamine receptor and mediates cellular responses to histamine. Functions as receptor for diazepines and various anesthetics, such as pentobarbital; these are bound at a separate allosteric effector binding site. Functions as ligand- gated chloride channel; Belongs to the ligand-gated ion channel (TC 1.A.9) family. Gamma-aminobutyric acid receptor (TC 1.A.9.5) subfamily. GABRG2 sub-subfamily
GABRA5	Gamma-aminobutyric acid receptor subunit alpha-5; GABA, the major inhibitory neurotransmitter in the vertebrate brain, mediates neuronal inhibition by binding to the GABA/benzodiazepine receptor and opening an integral chloride channel; Gamma-aminobutyric acid type A receptor subunits
GABRB3	Gamma-aminobutyric acid receptor subunit beta-3; Component of the heteropentameric receptor for GABA, the major inhibitory neurotransmitter in the vertebrate brain. Functions also as

histamine receptor and mediates cellular responses to histamine. Functions as receptor for diazepines and various anesthetics, such as pentobarbital; these are bound at a separate allosteric effector binding site. Functions as ligand- gated chloride channel; Belongs to the ligand-gated ion channel (TC 1.A.9) family. Gamma-aminobutyric acid receptor (TC 1.A.9.5) subfamily. GABRB3 sub-subfamily

GABRA5	Gamma-aminobutyric acid receptor subunit alpha-5; GABA, the major inhibitory neurotransmitter in the vertebrate brain, mediates neuronal inhibition by binding to the GABA/benzodiazepine receptor and opening an integral chloride channel; Gamma-aminobutyric acid type A receptor subunits
GABRB2	Gamma-aminobutyric acid receptor subunit beta-2; Component of the heteropentameric receptor for GABA, the major inhibitory neurotransmitter in the vertebrate brain. Functions also as histamine receptor and mediates cellular responses to histamine. Functions as receptor for diazepines and various anesthetics, such as pentobarbital; these are bound at a separate allosteric effector binding site. Functions as ligand- gated chloride channel; Gamma-aminobutyric acid type A receptor subunits
GABRD	Gamma-aminobutyric acid receptor subunit delta; GABA, the major inhibitory neurotransmitter in the vertebrate brain, mediates neuronal inhibition by binding to the GABA/benzodiazepine receptor and opening an integral chloride channel; Gamma-aminobutyric acid type A receptor subunits
STXBP1	Syntaxin-binding protein 1; May participate in the regulation of synaptic vesicle docking and fusion, possibly through interaction with GTP-binding proteins. Essential for neurotransmission and binds syntaxin, a component of the synaptic vesicle fusion machinery probably in a 1:1 ratio. Can interact with syntaxins 1, 2, and 3 but not syntaxin 4. May play a role in determining the specificity of intracellular fusion reactions
STX1B	Syntaxin-1B; Potentially involved in docking of synaptic vesicles at presynaptic active zones. May mediate Ca(2+)-regulation of exocytosis acrosomal reaction in sperm (By similarity); Belongs to the syntaxin family
CPLX1	Complexin-1/2; Complexin-1; Positively regulates a late step in exocytosis of various cytoplasmic vesicles, such as synaptic vesicles and other secretory vesicles. Organizes the SNAREs into a cross-linked zigzag topology that, when interposed between the vesicle and plasma membranes, is incompatible with fusion, thereby preventing SNAREs from releasing neurotransmitters until an action potential arrives at the synapse. Also involved in glucose-induced secretion of insulin by pancreatic beta-cells. Essential for motor behavior
SNAP29	Synaptosomal-associated protein 29; SNAREs, soluble N-ethylmaleimide-sensitive factor-attachment protein receptors, are essential proteins for fusion of cellular membranes. SNAREs localized on opposing membranes assemble to form a trans-SNARE complex, an extended, parallel four alpha-helical bundle that drives membrane fusion. SNAP29 is a SNARE involved in autophagy through the direct control of autophagosome membrane fusion with the lysososome membrane. Plays also a role in ciliogenesis by regulating membrane fusions; Belongs to the SNAP-25 family
GOSR2	Golgi SNAP receptor complex member 2; Involved in transport of proteins from the cis/medial-Golgi to the trans-Golgi network; Belongs to the GOSR2 family
DNAJC5	DnaJ homolog subfamily C member 5; Acts as a general chaperone in regulated exocytosis (By similarity). Acts as a co-chaperone for the SNARE protein SNAP-25 (By similarity). Involved in the calcium-mediated control of a late stage of exocytosis (By similarity). May have an important role in presynaptic function. May be involved in calcium-dependent neurotransmitter release at nerve endings (By similarity); DNAJ heat shock proteins
SYP	Synaptophysin; Possibly involved in structural functions as organizing other membrane components or in targeting the vesicles to the plasma membrane. Involved in the regulation of short-term and long-term synaptic plasticity (By similarity); Synaptophysins
NAPB	Beta-soluble NSF attachment protein; Required for vesicular transport between the endoplasmic reticulum and the Golgi apparatus
KCNA2	Potassium voltage-gated channel subfamily A member 2; Voltage-gated potassium channel that mediates transmembrane potassium transport in excitable membranes, primarily in the brain and the central nervous system, but also in the cardiovascular system. Prevents aberrant action potential firing and regulates neuronal output. Forms tetrameric potassium- selective channels through which potassium ions pass in accordance with their electrochemical gradient. The channel alternates between opened and closed conformations in response to the voltage difference across the membrane. Can form func [...]
KCNA1	Potassium voltage-gated channel subfamily A member 1; Voltage-gated potassium channel that mediates transmembrane potassium transport in excitable membranes, primarily in the brain and the central nervous system, but also in the kidney. Contributes to the regulation of the membrane potential and nerve signaling, and prevents neuronal hyperexcitability. Forms

	tetrameric potassium- selective channels through which potassium ions pass in accordance with their electrochemical gradient.
CNTNAP2	Contactin-associated protein-like 2; Required, with CNTNAP1, for radial and longitudinal organization of myelinated axons. Plays a role in the formation of functional distinct domains critical for saltatory conduction of nerve impulses in myelinated nerve fibers. Demarcates the juxtaparanodal region of the axo-glial junction; Belongs to the neurexin family
CNTN2	Contactin-2; In conjunction with another transmembrane protein, CNTNAP2, contributes to the organization of axonal domains at nodes of Ranvier by maintaining voltage-gated potassium channels at the juxtaparanodal region. May be involved in cell adhesion; Fibronectin type III domain containing
KCNQ2	Potassium voltage-gated channel subfamily KQT member 2; Associates with KCNQ3 to form a potassium channel with essentially identical properties to the channel underlying the native M-current, a slowly activating and deactivating potassium conductance which plays a critical role in determining the subthreshold electrical excitability of neurons as well as the responsiveness to synaptic inputs. Therefore, it is important in the regulation of neuronal excitability. KCNQ2/KCNQ3 current is blocked by linopirdine and XE991, and activated by the anticonvulsant retigabine.
KCNB1	Potassium voltage-gated channel subfamily B member 1; Voltage-gated potassium channel that mediates transmembrane potassium transport in excitable membranes, primarily in the brain, but also in the pancreas and cardiovascular system. Contributes to the regulation of the action potential (AP) repolarization, duration and frequency of repetitive AP firing in neurons, muscle cells and endocrine cells and plays a role in homeostatic attenuation of electrical excitability throughout the brain.
KCNQ3	Potassium voltage-gated channel subfamily KQT member 3; Associates with KCNQ2 or KCNQ5 to form a potassium channel with essentially identical properties to the channel underlying the native M-current, a slowly activating and deactivating potassium conductance which plays a critical role in determining the subthreshold electrical excitability of neurons as well as the responsiveness to synaptic inputs. Therefore, it is important in the regulation of neuronal excitability; Belongs to the potassium channel family. KQT (TC 1.A.1.15) subfamily. Kv7.3/KCNQ3 sub-subfamily
CACNA1A	Voltage-dependent P/Q-type calcium channel subunit alpha-1A; Voltage-sensitive calcium channels (VSCC) mediate the entry of calcium ions into excitable cells and are also involved in a variety of calcium-dependent processes, including muscle contraction, hormone or neurotransmitter release, gene expression, cell motility, cell division and cell death. The isoform alpha-1A gives rise to P and/or Q-type calcium currents.
CACNA1H	Voltage-dependent T-type calcium channel subunit alpha-1H; Voltage-sensitive calcium channels (VSCC) mediate the entry of calcium ions into excitable cells and are also involved in a variety of calcium-dependent processes, including muscle contraction, hormone or neurotransmitter release, gene expression, cell motility, cell division and cell death. The isoform alpha-1H gives rise to T-type calcium currents. T-type calcium channels belong to the "low-voltage activated (LVA)" group and are strongly blocked by nickel and mibefradil.
CACNA1E	Voltage-dependent R-type calcium channel subunit alpha-1E; Voltage-sensitive calcium channels (VSCC) mediate the entry of calcium ions into excitable cells and are also involved in a variety of calcium-dependent processes, including muscle contraction, hormone or neurotransmitter release, gene expression, cell motility, cell division and cell death. The isoform alpha-1E gives rise to R-type calcium currents. R-type calcium channels belong to the 'high-voltage activated' (HVA) group and are blocked by nickel, and partially by omega-agatoxin-IIIA (omega-Aga-IIIA)
CACNB4	Voltage-dependent L-type calcium channel subunit beta-4; The beta subunit of voltage-dependent calcium channels contributes to the function of the calcium channel by increasing peak calcium current, shifting the voltage dependencies of activation and inactivation, modulating G protein inhibition and controlling the alpha-1 subunit membrane targeting
RYR3	Ryanodine receptor 3; Calcium channel that mediates the release of Ca(2+) from the sarcoplasmic reticulum into the cytoplasm in muscle and thereby plays a role in triggering muscle contraction. May regulate Ca(2+) release by other calcium channels. Calcium channel that mediates Ca(2+)-induced Ca(2+) release from the endoplasmic reticulum in non-muscle cells. Contributes to cellular calcium ion homeostasis (By similarity). Plays a role in cellular calcium signaling; EF-hand domain containing
COG6	Conserved oligomeric Golgi complex subunit 6; Required for normal Golgi function; Components of oligomeric golgi complex
COG7	Conserved oligomeric Golgi complex subunit 7; Required for normal Golgi function; Components of oligomeric golgi complex

COG1	Conserved oligomeric Golgi complex subunit 1; Required for normal Golgi function; Components of oligomeric golgi complex
COG5	Conserved oligomeric Golgi complex subunit 5; Required for normal Golgi function; Belongs to the COG5 family
COG8	Conserved oligomeric Golgi complex subunit 8; Required for normal Golgi function; Components of oligomeric golgi complex
COG4	Conserved oligomeric Golgi complex subunit 4; Required for normal Golgi function. Plays a role in SNARE-pin assembly and Golgi-to-ER retrograde transport via its interaction with SCFD1; Belongs to the COG4 family.
GLI2	Zinc finger protein GLI2; Functions as transcription regulator in the hedgehog (Hh) pathway. Functions as transcriptional activator. May also function as transcriptional repressor (By similarity). Requires STK36 for full transcriptional activator activity. Required for normal embryonic development; Zinc fingers C2H2-type
GLI3	Transcriptional activator GLI3; Has a dual function as a transcriptional activator and a repressor of the sonic hedgehog (Shh) pathway, and plays a role in limb development. The full-length GLI3 form (GLI3FL) after phosphorylation and nuclear translocation, acts as an activator (GLI3A) while GLI3R, its C-terminally truncated form, acts as a repressor. A proper balance between the GLI3 activator and the repressor GLI3R, rather than the repressor gradient itself or the activator/repressor ratio gradient, specifies limb digit number and identity
PTCH1	Protein patched homolog 1; Acts as a receptor for sonic hedgehog (SHH), indian hedgehog (IHH) and desert hedgehog (DHH). Associates with the smoothened protein (SMO) to transduce the hedgehog's proteins signal. Seems to have a tumor suppressor function, as inactivation of this protein is probably a necessary, if not sufficient step for tumorigenesis; Belongs to the patched family
ZIC2	Zinc finger protein ZIC 2; Acts as a transcriptional activator or repressor. Plays important roles in the early stage of organogenesis of the CNS. Activates the transcription of the serotonin transporter SERT in uncrossed ipsilateral retinal ganglion cells (iRGCs) to refine eye-specific projections in primary visual targets. Its transcriptional activity is repressed by MDFIC. Involved in the formation of the ipsilateral retinal projection at the optic chiasm midline.
SHH	Sonic hedgehog protein; Sonic hedgehog protein: The C-terminal part of the sonic hedgehog protein precursor displays an autoproteolysis and a cholesterol transferase activity (By similarity). Both activities result in the cleavage of the full-length protein into two parts (ShhN and ShhC) followed by the covalent attachment of a cholesterol moiety to the C-terminal of the newly generated ShhN (By similarity). Both activities occur in the reticulum endoplasmic (By similarity). Once cleaved, ShhC is degraded in the endoplasmic reticulum (By similarity); Hedgehog signaling molecule family
CDON	Cell adhesion molecule-related/down-regulated by oncogenes; Component of a cell-surface receptor complex that mediates cell-cell interactions between muscle precursor cells. Promotes differentiation of myogenic cells (By similarity); Fibronectin type III domain containing
